# Occupational Risk Factors for Burnout Syndrome Among Healthcare Professionals: A Global Systematic Review and Meta-Analysis

**DOI:** 10.3390/ijerph21121583

**Published:** 2024-11-27

**Authors:** Sohrab Amiri, Nailah Mahmood, Halla Mustafa, Syed Fahad Javaid, Moien AB Khan

**Affiliations:** 1 Spiritual Health Research Center, Life Style Institute, Baqiyatallah University of Medical Sciences, Tehran 17166, Iran; rsr.amiri.s@bmsu.ac.ir; 2 Division of Health Research, Lancaster University, Lancaster LA1 4YW, UK; n.mahmood2@lancaster.ac.uk; 3 Health and Wellness Research Group, Department of Family Medicine, College of Medicine and Health Sciences, United Arab Emirates University, Al-Ain 15551, United Arab Emirates; h.mustafa@uaeu.ac.ae; 4 Health and Wellness Research Group, Department of Psychiatry, College of Medicine and Health Sciences, United Arab Emirates University, Al-Ain 15551, United Arab Emirates

**Keywords:** burnout syndrome, health professionals, occupational risk factors, meta-analysis, systematic review

## Abstract

Health professionals are disproportionately affected by burnout compared to other occupational groups. This study aims to systematically review and meta-analyze thirteen occupational risk factors related to burnout syndrome among health professionals globally. A comprehensive literature search was conducted in August 2023. The protocol was registered in The International Prospective Register of Systematic Reviews (PROSPERO), registration number CRD42023396081. Using a random-effects model, this meta-analysis assessed the association between occupational risk factors and burnout, reporting odds ratios (ORs) and 95% confidence intervals (CIs). The meta-analysis included 109 studies from diverse global locations. Key factors influencing burnout included workplace bullying, job stress, and poor communication, with protective factors such as supportive work environments, adequate staffing, and individual resilience. All risk factors examined showed a significant positive relationship with burnout incidence. Workplace bullying was strongly associated with increased burnout (OR 4.05–15.01, *p* < 0.001). Similarly, low job satisfaction and high job stress were strongly associated with burnout, with ORs of 5.05 (95% CI 3.88–6.56, *p* < 0.001) and 4.21 (95% CI 1.62–10.94, *p* = 0.003), respectively. The review findings highlight the importance of addressing these risk factors through enhanced supportive work environments and promoting personal resilience strategies.

## 1. Introduction

Burnout syndrome is a condition characterized by the inability to effectively manage work-related stress, leading to three key elements: feelings of exhaustion or energy depletion, increased mental distance or negative feelings towards one’s job, and reduced professional efficacy [1]. The study of burnout has been primarily driven by Maslach, who developed a widely used questionnaire that assesses burnout based on three components: emotional exhaustion (EE), depersonalization (D), and decreased personal accomplishment (PA) [2,3].

Burnout syndrome is a widespread phenomenon across various occupational settings [4], with a particularly important focus on the healthcare industry [5,6]. Studies have found a significant prevalence of burnout syndrome among healthcare professionals [5,6,7], with a global study reporting a prevalence rate of 67.0% [5]. Physicians are particularly susceptible to burnout, with a 15-fold higher risk compared to other professionals [8]. Emergency physicians, in particular, have been found to have high rates of burnout, with 88.6% experiencing medium to high emotional exhaustion and 82.8% experiencing medium to high depersonalization [9]. The global prevalence of burnout syndrome among nurses is 11.2% [10]. Healthcare professionals face unique stressors and challenges that contribute to burnout. Experiencing intense situations, working long hours, having a high patient load, and being exposed to trauma and suffering often all contribute to burnout [5,6,7]. Burnout syndrome negatively impacts various job dimensions, physical health, and productivity beyond the individual. Furthermore, burnout syndrome has been linked to a higher risk of medical errors, highlighting the importance of proactive measures. The COVID-19 pandemic has further exacerbated burnout syndrome among healthcare professionals, leading to a plethora of studies on this topic [11,12,13,14].

Various factors can contribute to burnout syndrome in health professionals, including age, marital status, lack of control over work, job seniority, work shifts, and work demands [15,16,17,18,19,20,21]. A broad range of studies have examined the risk factors of burnout syndrome in health professionals, and some of these factors have been examined [15,17,21,22,23,24,25,26,27].

Despite the large amount of literature about burnout syndrome among health professionals and a detailed review of studies that have examined occupational risk factors for burnout syndrome in this population, several issues emerged that provided the basis for this research. Firstly, no comprehensive global study has addressed occupational burnout syndrome risk factors. What has been done has mostly focused on investigating the prevalence of occupational burnout syndrome or has examined only some risk factors of occupational burnout. Identifying the underlying factors of burnout syndrome can increase insight and help make health policy more robust. In addition, we do not know which occupational risk factors contribute the most to burnout syndrome, in other words, the hierarchy of occupational risk factors for burnout syndrome in healthcare professionals.

In this study, thirteen occupational risk factors related to burnout syndrome are systematically reviewed and meta-analyzed, and a hierarchy of the most important causes are presented. This research, therefore, aims to provide the most comprehensive and complete analysis of occupational risk factors and burnout syndrome in health professionals.

## 2. Materials and Methods

### 2.1. Registration and Protocol

The research protocol was based on the Preferred Reporting Items for Systematic Reviews and Meta-Analyses (PRISMA) standards [28], as depicted in the PRISMA checklist (Supplementary File S1). The protocol was registered in The International Prospective Register of Systematic Reviews (PROSPERO), registration number CRD42023396081 (Supplementary File S2).

### 2.2. Eligibility Criteria

The inclusion criteria for the current study included the following: (1) The study population was health professionals. (2) Cross-sectional, cohort and case-control studies were eligible. (3) To reduce selection bias, a minimum sample size of 100 participants was considered for each study. These studies were not eligible: (1) Studies whose population was volunteers were not eligible. (2) Studies that did not report enough data to calculate the odds ratio and 95% confidence interval.

### 2.3. Information Sources

This research systematically searched three databases (PubMed, Web of Science, and Scopus) and manually searched one gray literature database (Google Scholar). To retrieve more studies, the references of similar studies were checked. For the systematic search, a keyword syntax was used for each database. The search in these databases was carried out until August 2023, and all the manuscripts were included from the beginning of their formation.

### 2.4. Search Strategy

This review was conducted following the PRISMA standards [29]. SA and MABK conducted each stage of the review procedure separately. Both authors individually evaluated each obtained record from the systematic and manual search using the predetermined keywords (Supplementary S3). In addition, they independently performed the risk of bias evaluation for each paper that was included (Supplementary S4). Disagreements between SA and MABK were addressed and resolved through consensus or by engaging a third reviewer, NM. Initially, data extraction was performed by one reviewer. However, this procedure was later modified to incorporate independent verification by the second author.

### 2.5. Selection Process

First, the studies were stored in a file and screened based on the title and abstract. All the authors participated in this process. After identifying potentially eligible studies, all their full texts were collected. In the process of screening the articles, all the authors worked independently. Regarding the final studies, the articles were screened interactively. In general, screening included four components: population, exposure, comparison, and outcomes.

### 2.6. Data Collection Process

Data was first extracted in August 2023. Each author synthesized a subset of eligible studies and extracted the necessary data. This process was independent, but, in the end, each mutually rechecked the extracted data from the other authors.

### 2.7. Data Items

The exposure variables in this research included a variety of occupational factors, including job demands, working hours, job stress, job strain, social support at work, job satisfaction, job control, job insecurity, workload, work–life imbalance, effort–reward imbalance, violence at work, and workplace bullying. The definitions of each of these were based on the definitions in the eligible articles. The outcome variable of interest in this study was burnout syndrome. The measurement of this variable was also based on the models briefly included in the Supplementary table (Supplementary S4).

### 2.8. Study Risk of Bias Assessment

In measuring the risk of bias, the Effective Public Health Practice Project Quality Assessment Tool [30,31] was used as a reliable tool that included four dimensions: selection bias, confounders bias, data collection method bias, and withdrawals and dropouts bias (Supplementary S5).

### 2.9. Effect Measures

In this research, the effect size used was an odds ratio and 95% confidence interval. The odds ratio and the standard error of the odds ratio were used to check for heterogeneity and publication bias.

### 2.10. Synthesis Methods

Eligible studies were able to calculate the odds ratio and 95% confidence interval; based on this, studies that reported the following indicators in the relationship between the exposure variable and the outcome were included in the meta-analysis: odds ratio and 95% confidence interval reported; or the Pearson correlation coefficient and sample size; or the mean, standard deviation, and sample size in the case and control groups. The data were converted to the odds ratio and 95% confidence interval by Comprehensive meta-analysis-3 software [32]. The standard error for the odd ratio was also calculated. This procedure was performed for all levels of exposure variables. In the following, the heterogeneity was checked using the heterogeneity chi-squared test and *I*^2^ [33,34]. Selection bias was assessed as part of the analyses using a funnel plot, Egger’s test, and the trim-and-fill method [35,36,37]. “The ‘trim and fill’ method aims both to identify and correct for funnel plot asymmetry arising from publication bias. The basis of the method is to (1) ‘trim’ (remove) the smaller studies causing funnel plot asymmetry, (2) use the trimmed funnel plot to estimate the true ‘centre’ of the funnel, then (3) replace the omitted studies and their missing ‘counterparts’ around the centre (filling)” [38]. “Egger’s test is commonly used to assess potential publication bias in a meta-analysis via funnel plot asymmetry (Egger’s test is a linear regression of the intervention effect estimates on their standard errors weighted by their inverse variance)” [38]. In the review of publication bias, the number of 10 studies was considered as a cut-off point [39].

## 3. Results

### 3.1. Study Selection

**Figure 1:**
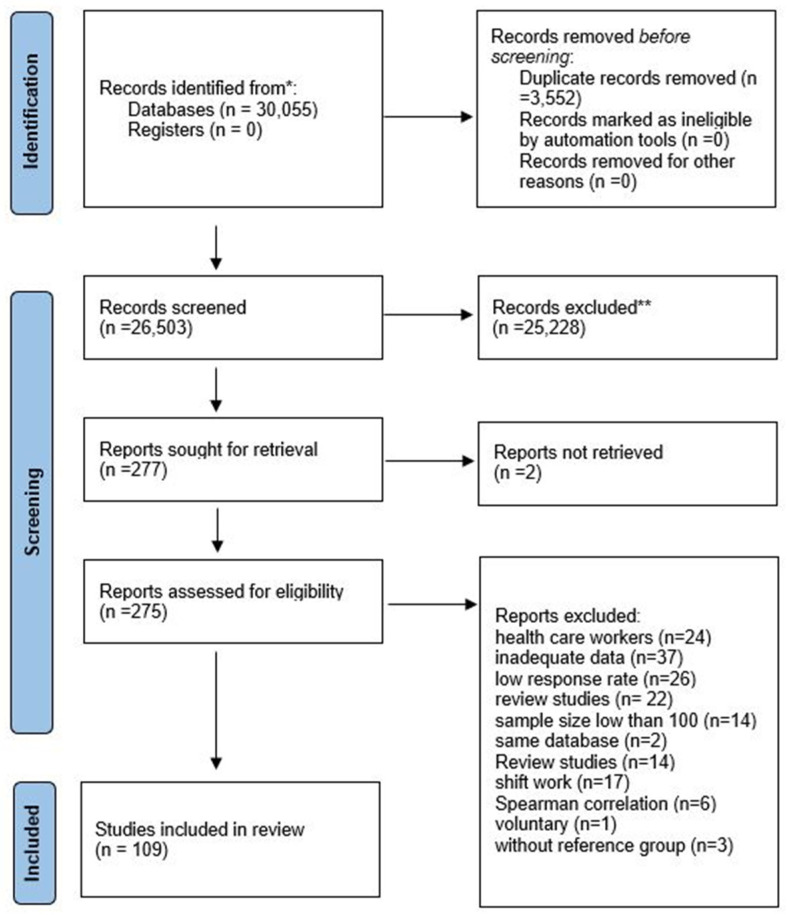
The PRISMA flow diagram. * The number of records identified from each database or register searched (rather than the total number across all databases/registers). ** Automation tools utilized to exclude records.

Figure 1 depicts the screening process for the studies. The studies were screened step by step. Finally, based on this study’s eligibility criteria, 109 studies [39,40,41,42,43,44,45,46,47,48,49,50,51,52,53,54,55,56,57,58,59,60,61,62,63,64,65,66,67,68,69,70,71,72,73,74,75,76,77,78,79,80,81,82,83,84,85,86,87,88,89,90,91,92,93,94,95,96,97,98,99,100,101,102,103,104,105,106,107,108,109,110,111,112,113,114,115,116,117,118,119,120,121,122,123,124,125,126,127,128,129,130,131,132,133,134,135,136,137,138,139,140,141,142,143,144,145,146,147] were selected, listed in the Supplementary table (Supplementary S4).

### 3.2. Study Characteristics

The characteristics of every study included in this research are listed in the supplementary table (Supplementary S4). These studies included a series of cross-sectional and longitudinal studies from all inhabited continents of the world. From the point of view of biographers, the age of the population studied in this meta-analysis was 18 years and older. In most of the studies, the population included both sexes. The two dominant populations in this study were nurses and physicians.

### 3.3. Risk of Bias in Studies

After the qualitative evaluation of the studies based on four dimensions, the results of this evaluation were included in the Supplementary table (Supplementary S4).

## 4. Results of Individual Studies

For each of the studies mentioned in this research, there was a range of data, including sample size, correlation coefficient, odds ratio, 95% confidence interval, and sample size, mean, and standard deviation in each group, which are listed in the Supplementary table (Supplementary S4).

## 5. Results of Syntheses

### 5.1. Long Working Hours and Burnout Syndrome

**Figure 2:**
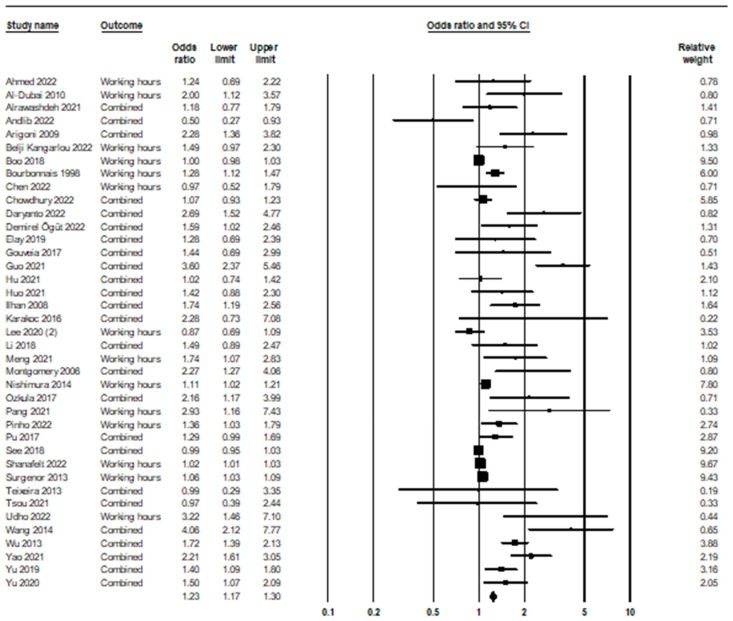
Long working hours and burnout syndrome [40,41,42,43,44,45,46,47,48,49,50,51,52,53,54,55,56,57,58,59,60,61,62,63,64,65,66,67,68,69,70,71,72,73,74,75,76,77].

In the relationship between long working hours and burnout syndrome, 39 studies were included in the meta-analysis, as shown in Figure 2. Based on the obtained result, the odds ratio was equal to 1.23, with a 95% confidence interval of 1.17–1.30 (*p* < 0.001; Z = 7.64; *I*^2^ = 83.21).

### 5.2. Job Demand and Burnout Syndrome

**Figure 3:**
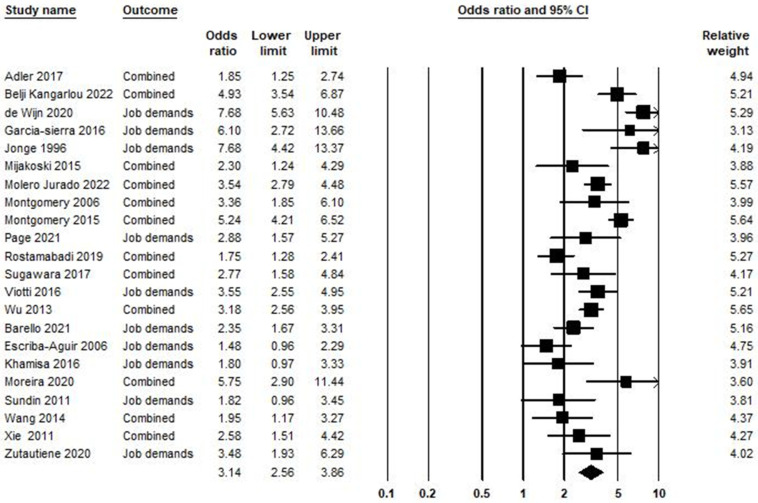
Job demand and burnout syndrome [61,72,73,78,79,80,81,82,83,84,85,86,87,88,89,90,91,92,93,94,95,96].

In the relationship between job demand and burnout syndrome, 22 studies were included in the meta-analysis, as shown in Figure 3. Based on the obtained result, the odds ratio was equal to 3.14, with a 95% confidence interval of 2.56–3.86 (*p* < 0.001; Z = 10.91; *I*^2^ = 82.43).

### 5.3. Workload and Burnout Syndrome

**Figure 4:**
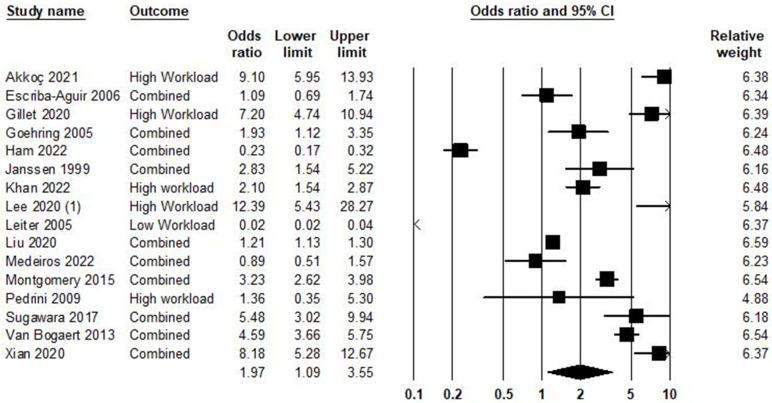
Workload and burnout syndrome [58,82,88,92,97,98,99,100,101,102,103,104,105,106,107,108].

In the relationship between workload and burnout syndrome, 16 studies were included in the meta-analysis, as shown in Figure 4. Based on the result, the odds ratio was equal to 1.97, with a 95% confidence interval of 1.09–3.55 (*p* = 0.024; Z = 2.26; *I*^2^ = 98.32).

### 5.4. Job Stress and Burnout Syndrome

**Figure 5:**
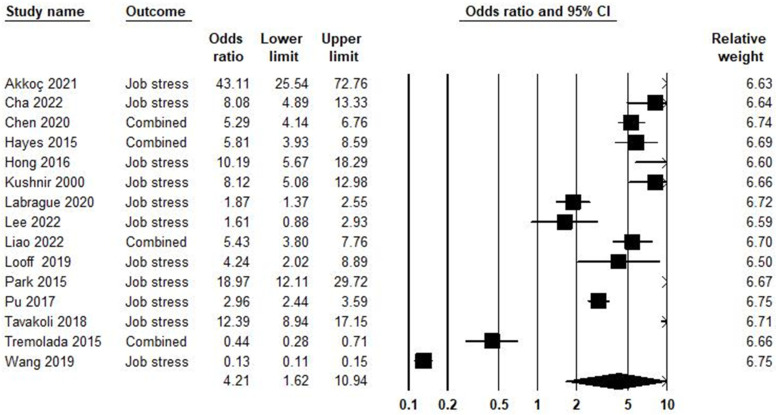
Job stress and burnout syndrome [66,97,109,110,111,112,113,114,115,116,117,118,119,120,121].

Job stress incorporates the psychological and emotional response to workplace challenges and conditions. Job stress is influenced not only by structural elements like demands and control but also by individual perceptions, coping mechanisms, and support systems, reflecting an individual’s subjective appraisal of stressors and their impact on well-being [148]. In the relationship between job stress and burnout syndrome, 15 studies were included in the meta-analysis, as shown in Figure 5. Based on the result, the odds ratio was equal to 4.21, with a 95% confidence interval of 1.62–10.94 (*p* = 0.003; Z = 2.95; *I*^2^ = 99.17).

### 5.5. Low Social Support at Work and Burnout Syndrome

**Figure 6:**
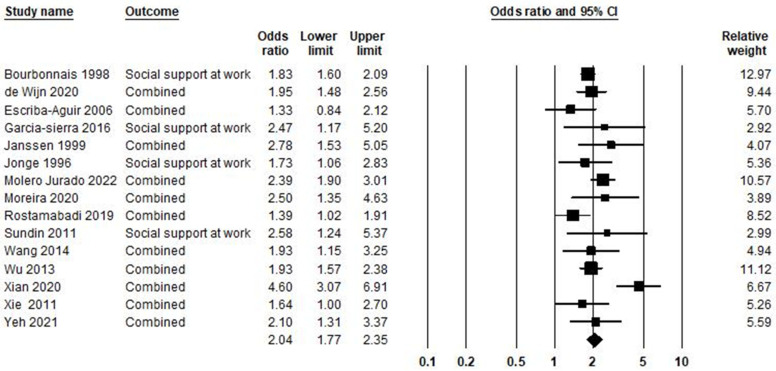
Low social support at work and burnout syndrome [46,72,73,81,82,83,84,87,89,91,93,95,101,108,122].

In the relationship between low social support at work and burnout syndrome, 15 studies were included in the meta-analysis, as shown in Figure 6. Based on the result, the odds ratio was equal to 2.04, with a 95% confidence interval of 1.77–2.35 (*p* < 0.001; Z = 9.86; I^2^ = 55.89).

### 5.6. Low Job Satisfaction and Burnout Syndrome

**Figure 7:**
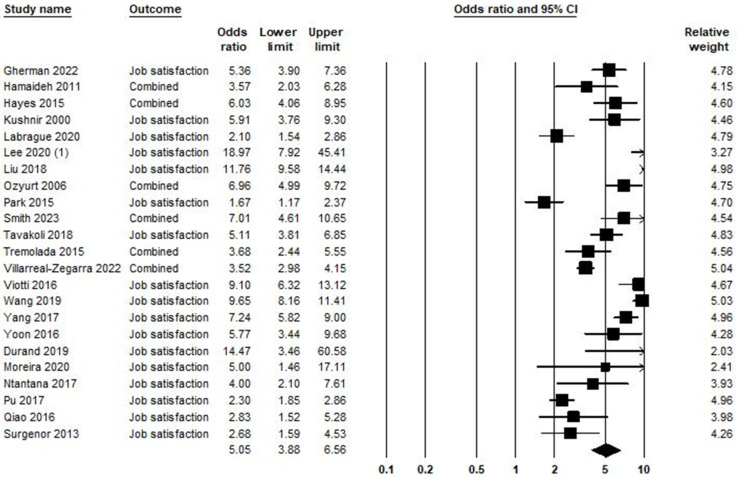
Low job satisfaction and burnout syndrome [58,59,66,68,89,94,112,114,115,118,119,120,121,123,124,125,126,127,128,129,130,131,132].

In the relationship between low job satisfaction and burnout syndrome, 23 studies were included in the meta-analysis, as shown in Figure 7. Based on the obtained result, the odds ratio was equal to 5.05, with a 95% confidence interval of 3.88–6.56 (*p* < 0.001; Z = 12.10; *I*^2^ = 92.89).

### 5.7. Job Control and Burnout Syndrome

**Figure 8:**
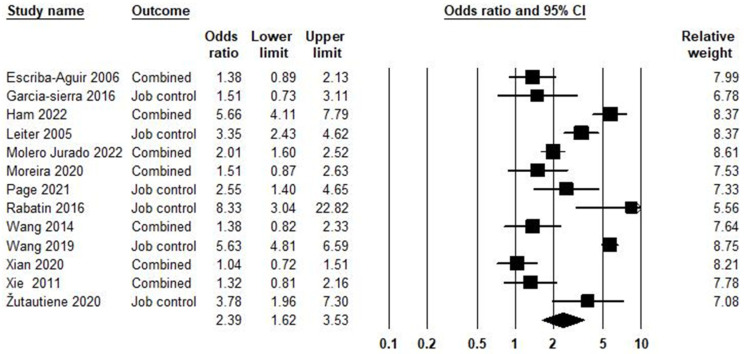
Job control and burnout syndrome [72,82,83,87,89,90,95,96,100,103,108,121,135].

Job control is one of the components of job strain. Based on Karasek’s job demands–control model, job strain is the combination of high job demands with low job control [4,133,134]. In the relationship between job control and burnout syndrome, 13 studies were included in the meta-analysis, as shown in Figure 8. Based on the obtained result, the odds ratio was equal to 2.39, with a 95% confidence interval of 1.62–3.53 (*p* < 0.001; Z = 4.39; *I*^2^ = 92.71%).

### 5.8. Work–Life Imbalance and Burnout Syndrome

**Figure 9:**
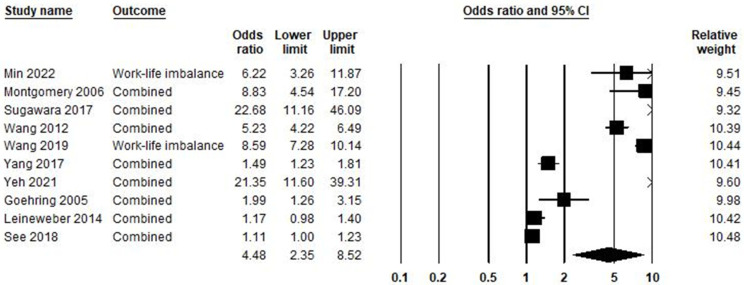
Work–life imbalance and burnout syndrome [61,67,92,99,121,122,131,136,137,138].

In the relationship between work–life imbalance and burnout syndrome, ten studies were included in the meta-analysis, as shown in Figure 9. Based on the result, the odds ratio was equal to 4.48, with a 95% confidence interval of 2.35–8.52 (*p* < 0.001; Z = 4.56; *I*^2^ = 98.65%).

### 5.9. Effort–Reward Imbalance, Other Stressors, and Burnout Syndrome

**Figure 10:**
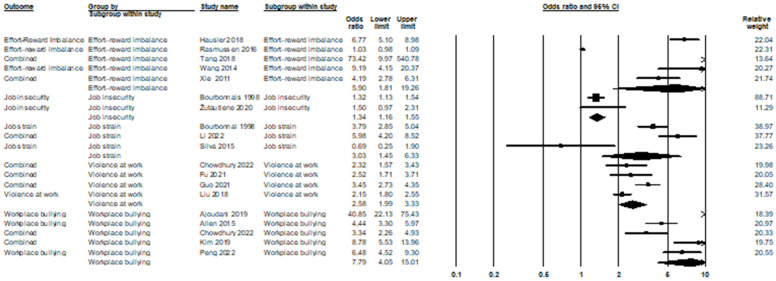
Effort–reward imbalance [72,95,139,140], job insecurity [46,96,141], job strain [46,141,142], violence at work [48,53,143,144], workplace bullying [48,145,146,149], and burnout syndrome.

Effort–reward imbalance is a mismatch between high efforts spent and low rewards received at work. In the relationship between effort–reward imbalance and burnout syndrome, five studies were included in the meta-analysis, and the odds ratio was equal to 5.90, with a 95% confidence interval of 1.81–19.26 (*p* = 0.003; Z = 2.94; *I*^2^ = 98.38%). In the relationship between job insecurity and burnout syndrome, two studies were included in the meta-analysis; the odds ratio was equal to 1.34, with a 95% confidence interval of 1.16–1.55 (*p* < 0.001; Z = 3.93; *I*^2^ = 0%). Job strain, a related yet distinct concept from job stress, refers to the structural imbalance between job demands and the degree of control an individual has over their work [148]. While job strain is often an objective measure based on workplace conditions, job stress is subjective and varies according to personal resilience and environmental factors [148]. In the relationship between job strain and burnout syndrome, three studies were included in the meta-analysis; the odds ratio was equal to 3.03, with a 95% confidence interval of 1.45–6.33 (*p* = 0.003; Z = 2.95; *I*^2^ = 87.93%). In the relationship between violence at work and burnout syndrome, four studies were included in the meta-analysis; the odds ratio was equal to 2.58, with a 95% confidence interval of 1.99–3.33 (*p* < 0.001; Z = 7.18; *I*^2^ = 71.41%). In the relationship between workplace bullying and burnout syndrome, five studies were included in the meta-analysis; the odds ratio was equal to 7.79, with a 95% confidence interval of 4.05–15.01 (*p* < 0.001; Z = 6.14; *I*^2^ = 92.45%), The relationship between these stressors and burnout syndrome is depicted in Figure 10.

### 5.10. Publication Bias and Heterogeneity

The publication bias in the association between long working hours and burnout syndrome is depicted in Supplementary Figure S1. The Egger test (*p* < 0.001) showed publication bias. The trim-and-fill [37] imputed 15 studies. Heterogeneity across studies was equal to *I^2^* = 83.21%; this means high heterogeneity [150], and the heterogeneity of chi-square was equal to 226.34 (d.f = 38; *p* < 0.001).

The publication bias in the association between job demand and burnout syndrome is shown in Supplementary Figure S2 The Egger test (*p* = 0.285) did not show publication bias. The trim-and-fill [37] has not imputed any study. Heterogeneity across studies was equal to *I*^2^ = 82.43%; this means high heterogeneity [150], and the heterogeneity of chi-square was equal to 119.52 (d.f = 21; *p* < 0.001).

Supplementary Figure S3 demonstrates publication bias in the association between workload and burnout syndrome (Figure S3). The Egger test (*p* = 0.433) did not show publication bias. The trim-and-fill [37] imputed five studies. Heterogeneity across studies was equal to *I^2^* = 98.32%; this means high heterogeneity [150], and the heterogeneity of chi-square was equal to 893.78 (d.f = 15; *p* < 0.001).

The publication bias in the association between job stress and burnout syndrome is shown in Supplementary Figure S4. The Egger test (*p* = 0.020) showed publication bias. The trim-and-fill [37] imputed four studies. Heterogeneity across studies was equal to *I^2^* = 99.17%; this means high heterogeneity [150], and the heterogeneity of chi-square was equal to 1696.69 (d.f = 14; *p* < 0.001).

Supplementary Figure S5 examines the publication bias associated with low social support at work and burnout syndrome (Figure S5). The Egger test (*p* = 0.379) did not show publication bias. The trim-and-fill [37] has not been imputed in any study. Heterogeneity across studies was equal to *I^2^* = 55.89%; this means medium heterogeneity [150], and the heterogeneity of chi-square was equal to 31.74 (d.f = 14; *p* = 0.004).

The publication bias in the association between low job satisfaction and burnout syndrome is shown in Supplementary Figure S6. The Egger test (*p* = 0.646) did not show publication bias. The trim-and-fill [37] imputed two studies. Heterogeneity across studies was equal to *I^2^* = 92.89%, which means high heterogeneity [150], and the heterogeneity of chi-square was equal to 309.305 (d.f = 22; *p* < 0.001).

The publication bias in the association between job control and burnout syndrome is demonstrated in Supplementary Figure S7. The Egger test (*p* = 0.081) showed publication bias. The trim-and-fill [37] has not imputed any study. Heterogeneity across studies was equal to *I^2^* = 92.71%, which means high heterogeneity [150], and the heterogeneity of chi-square was equal to 164.601 (d.f = 12; *p* < 0.001).

Supplementary Figure S8 depicts the publication bias associated with work–family imbalance and burnout syndrome (Figure S8). The Egger test (*p* = 0.124) did not show publication bias. The trim-and-fill [37] has not imputed any study. Heterogeneity across studies was equal to *I^2^* = 98.65%; this means high heterogeneity [150], and the heterogeneity of chi-square was equal to 665.10 (d.f = 9; *p* < 0.001).

## 6. Discussion

The prevalence of burnout varies significantly across different healthcare professions and settings, ranging from 3.3% to 64.0% [9,15,70,102,117,119,121,130,151,152,153]. These findings are consistent with previous studies reporting varying burnout rates across professions, highlighting demographic factors such as age, gender, and marital status as significant contributors to burnout levels [82,91,122]

In previous studies, health professionals are at high risk of burnout syndrome due to emotional pressure and a work environment characterized by stress caused by dealing with patients and the deaths of patients [154,155]. Another prominent factor influencing burnout in healthcare professionals is secondary traumatic stress (STS), a condition arising from indirect exposure to trauma, such as through repeated encounters with patient suffering and death [156]. STS mimics symptoms of direct trauma, including anxiety, detachment, and intrusive thoughts, contributing to an intensified emotional toll on healthcare workers [157]. Evidence suggests that professionals engaged in frequent end-of-life care experience heightened STS, which exacerbates burnout [158]. Other work-related factors, including workload, job stress, temporary work contracts, conflicts, ethical decision-making, and occupational stress, have been identified as significant contributors to burnout [14,70,72,94,100,107,119,120,138,159,160,161]. This is supported by studies examining the impact of job demands, lack of support, and workplace bullying on exacerbating burnout levels [78,80]. However, while these studies shed light on the adverse effects of a hostile workplace environment, the complex interplay between individual resilience and organizational culture deserves further exploration. Nevertheless, these findings underscore the importance of workload management strategies and the need for organizations to implement measures to alleviate excessive work demands.

Similarly, workplace bullying, effort–reward imbalance, and low job satisfaction were among the factors leading to burnout syndrome. As a result, these factors increased the risk of job burnout by nearly eight times, nearly six times, and almost five times, respectively. Long working hours and lack of job security were factors at the bottom of the hierarchy of occupational risk factors for burnout syndrome. While extended working hours and employment stability are relevant, they are often secondary to more significant stressors, such as workload demands and perceived lack of control [162,163]. Limited autonomy and decision-making power weigh more heavily on the personal well-being and efficacy of healthcare professionals and drive burnout more strongly than total work hours or worries about job security [163,164]. These findings highlight the crucial role these occupational risk factors play in the development of burnout syndrome among health professionals. Addressing these workplace stressors is essential for fostering a supportive and safe work environment conducive to staff well-being.

Burnout syndrome is considered a psychological phenomenon [164]. Therefore, many factors may influence its psychological dimension. As a result, it comes as no surprise that occupational risk factors contribute to burnout syndrome. Various mechanisms can influence burnout syndrome depending on the investigated occupational risk factor. Studies have shown that occupational risk factors are associated with an increased risk of depression, which is an important underlying factor in burnout syndrome [165,166,167,168,169,170]. It is a vicious cycle and can lead to job inefficiency, reduced income, and sick leave, aggravating job burnout [171,172]. Burnout syndrome in health professionals is a consequence of occupational risk factors and can negatively affect various job dimensions. Also, burnout syndrome reduces physical health and productivity [173,174,175]. In contrast, protective factors against burnout include supportive work environments, social support, healthy lifestyles, and adequate coping mechanisms, which are crucial in mitigating burnout levels [71,94,107,119,120,121,131]. Supportive leadership, adequate staffing, and good work–family balance have also been identified as protective factors [60,136,176,177]. This comprehensive approach is supported by the work of Lee (2022), Peng et al. (2022), and Silva et al. (2015), who found that resilience, communication competence, and social support significantly reduce burnout levels [116,142,149]. These findings highlight the potential for resilience-building interventions and organizational support structures to mitigate burnout and promote staff well-being.

Research on burnout syndrome among health professionals has increased the importance of awareness about the mental health and well-being of this population. Based on this, several countries, notably Sweden, Canada, the United Kingdom, and the United States, have implemented policies to address burnout among healthcare professionals by improving workplace conditions, promoting peer mentoring, increasing mental health resources, and implementing systemic reforms [163,178,179,180]. Health professionals must pay more attention to burnout syndrome since it is associated with a higher risk of medical errors [181,182].

The findings from this work have important clinical implications for healthcare professionals. To promote healthcare professionals’ mental health and well-being, healthcare organizations and policymakers must address the identified occupational risk factors. Health professionals are at risk of burnout syndrome because of emotional pressure and a stressful work environment. Interventions like enhanced workplace flexibility, staff counseling services, resilience training, and peer-based support programs should be implemented to help healthcare professionals cope with the emotional demands of their jobs and provide them with emotional support and coping strategies. Other factors that contribute to burnout syndrome are workplace bullying, effort–reward imbalance, and low job satisfaction. Healthcare organizations must establish policies and mechanisms to prevent workplace harassment and bullying like anonymous reporting lines, ombudspersons, and leadership accountability systems. Healthcare professionals should be rewarded fairly and appropriately for their efforts with performance-based rewards, career advancement opportunities and enhanced wellness programs like family support services. There must exist opportunities for them to develop professionally and achieve work–life balance like access to continuous education programs, research and innovation grants, and soft-skills development initiatives.

Long working hours and job insecurity negatively impact burnout syndrome. To mitigate these risk factors, healthcare organizations should optimize work schedules, implement strategies to manage workload efficiently, and provide job security measures including transparent job progression schemes, robust mentorship, streamlined communication, and task sharing and delegation systems. This study also addresses the psychological dimension of burnout syndrome. As occupational risk factors can contribute to depression, mental health support services should be integrated into healthcare settings. Counselling, psychoeducation, and resilience-building programs should be provided regularly to support mental health for healthcare professionals.

This study entails a comprehensive meta-analysis of occupational risk factors for burnout syndrome. This research has limitations. Although most of the included studies used the same scale to measure burnout syndrome, the difference between the scales is a source of heterogeneity in this study. This study’s results may have been influenced by the heterogeneity in the results of most of the studies included in this study. There were insufficient studies for each of the occupational and gender risk factors to analyze subgroups. Since most of the studies in this research were cross-sectional, causal relationships cannot be drawn.

## 7. Conclusions

This systematic review and meta-analysis demonstrated a significant association between various occupational risk factors and burnout syndrome among health professionals. Bullying at work, an imbalance between effort and reward, and low job satisfaction were found to be the highest risk factors for burnout syndrome. Professionals should be made aware of burnout syndrome, and policies should be implemented to examine their mental health, specifically burnout syndrome. Burnout syndrome should be prioritized in health-related policies due to its potential consequences, including medical errors.

## Data Availability

The raw data supporting the conclusions of this article will be made available by the authors on request.

## Supplementary Materials

The following supporting information can be downloaded at: https://www.mdpi.com/article/10.3390/ijerph21121583/s1, Supplementary File S1: PRISMA checklist; Supplementary File S2: Study Protocol; Supplementary File S3: Keywords used for PubMed, Scopus, and Web of Science search; Supplementary File S4: Characteristics of the studies included in the analyses; Supplementary File S5: Quality assessment framework; Figure S1: Publication bias in the association between long working hours and burnout syndrome; Figure S2: Publication bias in the association between job demand and burnout syndrome; Figure S3: Publication bias in the association between workload and burnout syndrome; Figure S4: Publication bias in the association between job stress and burnout syndrome; Figure S5: Publication bias in the association between low social support at work and burnout syndrome; Figure S6: Publication bias in the association between low job satisfaction and burnout syndrome; Figure S7: Publication bias in the association between job control and burnout syndrome; Figure S8: Publication bias in the association between work–family imbalance and burnout syndrome.
