# Supplementary material for: Occupational Risk Factors for Burnout Syndrome Among Healthcare Professionals: A Global Systematic Review and Meta-Analysis
Source: Int J Environ Res Public Health. 2024 Nov 27;21(12):1583. doi: 10.3390/ijerph21121583 (PMC11675210; doi:10.3390/ijerph21121583)
Supplement: Supplementary file 1 [file ijerph-21-01583-s001.zip › Supplementary File S2.pdf]

Systematic review

A list of fields that can be edited in an update can be found here

1. * Review title.

Give the title of the review in English

Occupational risk factors for burnout syndrome in health professionals: a systematic review and meta-analysis

2. Original language title.

For reviews in languages other than English, give the title in the original language. This will be displayed with the English language title.

3. * Anticipated or actual start date.

Give the date the systematic review started or is expected to start.

01/01/2023

4. * Anticipated completion date.

Give the date by which the review is expected to be completed.

31/05/2023

5. * Stage of review at time of this submission.

This field uses answers to initial screening questions. It cannot be edited until after registration.

Tick the boxes to show which review tasks have been started and which have been completed.

Update this field each time any amendments are made to a published record.

The review has not yet started: No

Review stage	Started	Completed
Preliminary searches	Yes	No
Piloting of the study selection process	Yes	No
Formal screening of search results against eligibility criteria	No	No
Data extraction	No	No
Risk of bias (quality) assessment	No	No
Data analysis	No	No

Provide any other relevant information about the stage of the review here.

6. * Named contact.

The named contact is the guarantor for the accuracy of the information in the register record. This may be any member of the review team.

Moien Khan

Email salutation (e.g. "Dr Smith" or "Joanne") for correspondence:

Dr Khan

7. * Named contact email.

Give the electronic email address of the named contact.

moien.khan@nhs.net

8. Named contact address

Give the full institutional/organisational postal address for the named contact.

425 A Ewell Road, Surbiton, KT6 7ES

9. Named contact phone number.

Give the telephone number for the named contact, including international dialling code.

07738380105

10. * Organisational affiliation of the review.

Full title of the organisational affiliations for this review and website address if available. This field may be

completed as 'None' if the review is not affiliated to any organisation.

NHS

Organisation web address:

11. * Review team members and their organisational affiliations.

Give the personal details and the organisational affiliations of each member of the review team. Affiliation refers to groups or organisations to which review team members belong. **NOTE: email and country now MUST be entered for each person, unless you are amending a published record. PLEASE USE AN INSTITUTIONAL EMAIL ADDRESS IF POSSIBLE.**

Assistant/Associate Professor Moien Khan. NHS
Assistant/Associate Professor Sohrab Amiri. Baqiyatallah University of Medical Sciences
Assistant/Associate Professor Iffat Elbarazi. United Arab Emirates University
Halla Mustafa. United Arab emirates University

12. * Funding sources/sponsors.

Details of the individuals, organizations, groups, companies or other legal entities who have funded or sponsored the review.

None

Grant number(s)

State the funder, grant or award number and the date of award

13. * Conflicts of interest.

List actual or perceived conflicts of interest (financial or academic).

None

14. Collaborators.

Give the name and affiliation of any individuals or organisations who are working on the review but who are not listed as review team members. **NOTE: email and country must be completed for each person, unless you are amending a published record.**

15. * Review question.

State the review question(s) clearly and precisely. It may be appropriate to break very broad questions down into a series of related more specific questions. Questions may be framed or refined using PI(E)COS or similar where relevant.

In this research, the aim is to systematically review and meta-analyze occupational factors related to burnout

syndrome in health professionals.

Secondary objectives:

1. The relationship between workload and burnout syndrome is investigated.

2. The relationship between working hours and burnout syndrome is investigated.

3. The relationship between job strain and burnout syndrome is investigated.

4. The relationship between supervisor support and burnout syndrome is investigated.

5. The relationship between work family conflict and burnout syndrome is investigated.

16. * Searches.

State the sources that will be searched (e.g. Medline). Give the search dates, and any restrictions (e.g. language or publication date). Do NOT enter the full search strategy (it may be provided as a link or attachment below.)

In this research, three databases (PubMed, Web of Science, Scopus) will be systematically searched and one gray literature database (Google Scholar) will be manually searched. Also, to retrieve more studies, the references of similar studies will also be checked="checked" value="1". For systematic search, a keyword syntax will be used for each of the databases. Starting the search for studies will not have a time limit.

17. URL to search strategy.

Upload a file with your search strategy, or an example of a search strategy for a specific database, (including the keywords) in pdf or word format. In doing so you are consenting to the file being made publicly accessible. Or provide a URL or link to the strategy. Do NOT provide links to your search **results**.

Alternatively, upload your search strategy to CRD in pdf format. Please note that by doing so you are consenting to the file being made publicly accessible.

Do not make this file publicly available until the review is complete

18. * Condition or domain being studied.

Give a short description of the disease, condition or healthcare domain being studied in your systematic review.

Burnout syndrome has been receiving attention among health professionals, especially with the start of the covid-19 pandemic and its effects on the health system, this issue has become increasingly important. However, an important part of burnout syndrome can be caused by occupational risk factors. Therefore, this area needs more attention.

19. * Participants/population.

Specify the participants or populations being studied in the review. The preferred format includes details of both inclusion and exclusion criteria.

The study population in this research is all active health professionals. The outcome variable is burnout syndrome. Also, exposure variables include occupational risk factors. Studies published and available in English will be eligible. Observational studies, both cross-sectional and cohort, will be eligible.

20. * Intervention(s), exposure(s).

Give full and clear descriptions or definitions of the interventions or the exposures to be reviewed. The preferred format includes details of both inclusion and exclusion criteria.

Also, exposure variables include occupational risk factors.

21. * Comparator(s)/control.

Where relevant, give details of the alternatives against which the intervention/exposure will be compared (e.g. another intervention or a non-exposed control group). The preferred format includes details of both inclusion and exclusion criteria.

Not applicable

22. * Types of study to be included.

Give details of the study designs (e.g. RCT) that are eligible for inclusion in the review. The preferred format includes both inclusion and exclusion criteria. If there are no restrictions on the types of study, this should be stated.

All observational studies, including cross-sectional studies and cohort studies, were eligible for inclusion in this study. Review studies, intervention studies, and case studies were not eligible.

23. Context.

Give summary details of the setting or other relevant characteristics, which help define the inclusion or exclusion criteria.

Studies published in English and conducted on health professionals will be eligible.

24. * Main outcome(s).

Give the pre-specified main (most important) outcomes of the review, including details of how the outcome is defined and measured and when these measurement are made, if these are part of the review inclusion criteria.

The outcome studied in the present study was burnout syndrome. All burnout subscales will also be examined.

Measures of effect

Please specify the effect measure(s) for you main outcome(s) e.g. relative risks, odds ratios, risk difference, and/or 'number needed to treat.

The effect size used in this research will be the odd ratio and 95% confidence interval.

25. * Additional outcome(s).

List the pre-specified additional outcomes of the review, with a similar level of detail to that required for main outcomes. Where there are no additional outcomes please state 'None' or 'Not applicable' as appropriate to the review

Types of health professionals, gender differences, types of occupational risk factors and types of burnout syndrome will be investigated.

Measures of effect

Please specify the effect measure(s) for you additional outcome(s) e.g. relative risks, odds ratios, risk difference, and/or 'number needed to treat.

In this research, the effect size used was odd ratio and 95% confidence interval. The odds ratio and the standard error of the odds ratio will also be used to check for heterogeneity and publication bias.

26. * Data extraction (selection and coding).

Describe how studies will be selected for inclusion. State what data will be extracted or obtained. State how this will be done and recorded.

Study Selection:
After collecting the studies in the software, the manuscript titles will be screened. Duplicate studies will be excluded. Abstracts are then screened. Finally, the final studies will be identified based on the entry and exit criteria.

Data extraction:

Data extraction will be done independently and any disagreements will be resolved through discussion. It will include information extracted from demographic characteristics to results related to effect size.

27. * Risk of bias (quality) assessment.

State which characteristics of the studies will be assessed and/or any formal risk of bias/quality assessment tools that will be used.

The qualitative assessment of the studies of this research was done in five dimensions according to Effective Public Health Practice Project Quality Assessment Tool.

28. * Strategy for data synthesis.

Describe the methods you plan to use to synthesise data. This **must not be generic text** but should be **specific to your review** and describe how the proposed approach will be applied to your data. If meta-analysis is planned, describe the models to be used, methods to explore statistical heterogeneity, and software package to be used.

All data extracted from eligible studies will be converted to odds ratio and 95% confidence interval. In studies that did not report the odds ratio, the correlation coefficient will be used to calculate the odds ratio. Finally,

studies will be pooled based on the random effects method. The heterogeneity tests were the Q test and I^2, I^2 has different interpretations, including the low, medium, and high heterogeneity. Publication bias evaluation was done with funnel plots, Egger's test, and the Trim and fill.

29. * Analysis of subgroups or subsets.

State any planned investigation of 'subgroups'. Be clear and specific about which type of study or participant will be included in each group or covariate investigated. State the planned analytic approach.

Sub-group meta-analysis including, sex, health professionals, and burnout scales.

30. * Type and method of review.

Select the type of review, review method and health area from the lists below.

Type of review

Cost effectiveness

No

Diagnostic

No

Epidemiologic

No

Individual patient data (IPD) meta-analysis

No

Intervention

No

Living systematic review

No

Meta-analysis

Yes

Methodology

No

Narrative synthesis

No

Network meta-analysis

No

Pre-clinical

No

Prevention

No

Prognostic

No

Prospective meta-analysis (PMA)

No

Review of reviews

No

Service delivery

No

Synthesis of qualitative studies

No

Systematic review

Yes

Other

No

Health area of the review

Alcohol/substance misuse/abuse

No

Blood and immune system

No

Cancer

No

Cardiovascular

No

Care of the elderly

No

Child health

No

Complementary therapies

No

COVID-19

Yes

For COVID-19 registrations please tick all categories that apply. Doing so will enable your record to appear in area-specific searches

Chinese medicine
Diagnosis
Epidemiological

Genetics
Health impacts
Immunity
Long COVID
Mental health
PPE
Prognosis
Public health intervention
Rehabilitation
Service delivery
Transmission
Treatments
Vaccines
Other

Crime and justice

No

Dental

Yes

Digestive system

No

Ear, nose and throat

No

Education

No

Endocrine and metabolic disorders

No

Eye disorders

No

General interest

No

Genetics

No

Health inequalities/health equity

No

Infections and infestations

No

International development

No

Mental health and behavioural conditions

No

Musculoskeletal
No

Neurological
No

Nursing
Yes

Obstetrics and gynaecology
No

Oral health
No

Palliative care
No

Perioperative care
No

Physiotherapy
Yes

Pregnancy and childbirth
No

Public health (including social determinants of health)
Yes

Rehabilitation
No

Respiratory disorders
No

Service delivery
No

Skin disorders
No

Social care
No

Surgery
No

Tropical Medicine
No

Urological
No

Wounds, injuries and accidents

No

Violence and abuse

No

31. Language.

Select each language individually to add it to the list below, use the bin icon to remove any added in error.

English

There is not an English language summary

32. * Country.

Select the country in which the review is being carried out. For multi-national collaborations select all the countries involved.

England

33. Other registration details.

Name any other organisation where the systematic review title or protocol is registered (e.g. Campbell, or The Joanna Briggs Institute) together with any unique identification number assigned by them. If extracted data will be stored and made available through a repository such as the Systematic Review Data Repository (SRDR), details and a link should be included here. If none, leave blank.

34. Reference and/or URL for published protocol.

If the protocol for this review is published provide details (authors, title and journal details, preferably in Vancouver format)

Add web link to the published protocol.

Or, upload your published protocol here in pdf format. Note that the upload will be publicly accessible.

No I do not make this file publicly available until the review is complete

Please note that the information required in the PROSPERO registration form must be completed in full even if access to a protocol is given.

35. Dissemination plans.

Do you intend to publish the review on completion?

Yes

Give brief details of plans for communicating review findings.?

36. Keywords.

Give words or phrases that best describe the review. Separate keywords with a semicolon or new line. Keywords help PROSPERO users find your review (keywords do not appear in the public record but are included in searches). Be as specific and precise as possible. Avoid acronyms and abbreviations unless these are in wide use.

Burnout syndrome, Occupational risk factor, Health professionals, Systematic review, Meta-analysis

37. Details of any existing review of the same topic by the same authors.

If you are registering an update of an existing review give details of the earlier versions and include a full bibliographic reference, if available.

38. * Current review status.

Update review status when the review is completed and when it is published.New registrations must be ongoing so this field is not editable for initial submission.

Please provide anticipated publication date

Review_Ongoing

39. Any additional information.

Provide any other information relevant to the registration of this review.

40. Details of final report/publication(s) or preprints if available.

Leave empty until publication details are available OR you have a link to a preprint (NOTE: this field is not editable for initial submission). List authors, title and journal details preferably in Vancouver format.

Give the link to the published review or preprint.
