# Supplementary material for: Occupational Risk Factors for Burnout Syndrome Among Healthcare Professionals: A Global Systematic Review and Meta-Analysis
Source: Int J Environ Res Public Health. 2024 Nov 27;21(12):1583. doi: 10.3390/ijerph21121583 (PMC11675210; doi:10.3390/ijerph21121583)
Supplement: Supplementary file 1 [file ijerph-21-01583-s001.zip › Supplementary File S3.pdf]

Appendix S3: Keywords used for PubMed, Scopus, and Web of Science search

Search	Query
PubMed	11,808
#1	Burnout, Psychological [Mesh] OR Burnout, Psychological [Text Word] OR Psychological Burnout [Text Word] OR Burnout Syndrome [Text Word] OR Burnout, Professional [Text Word] OR Burnout, Professional [Text Word] OR Occupational Burnout [Text Word] OR Career Burnout [Text Word] OR emotional exhaustion [Text Word] OR physical exhaustion [Text Word] OR Job burnout [Text Word] OR Depersonalisation [Mesh] OR Depersonalisation [Text Word]
#2	Job Strain [Text Word] OR Job demands [Text Word] OR job control [Text Word] OR work strain [Text Word] OR Occupational Stresses [Mesh] OR Occupational Stresses [Text Word] OR Job Stress [Mesh] OR Job Stress [Text Word] OR Professional Stress [Mesh] OR Professional Stress [Text Word] OR Workplace Stress [Mesh] OR Workplace Stress [Text Word] OR Work long hours [Text Word] OR Long working hours [Text Word] OR working hours [Text Word] OR Heavy overtime work [Text Word] OR Overtime work [Text Word] OR Occupational Factors [Text Word] OR Work Conditions [Text Word] OR Risk Factors [Text Word] OR Occupational Stress [Mesh] OR Occupational Stress [Text Word] OR Workplace Bullying [Text Word] OR Job-related [Text Word] OR Occupational environment [Text Word] OR Professional environment [Text Word] OR Workplace Abuses [Text Word] OR Workplace [Text Word] OR effort reward imbalance [Text Word] OR Shift Work Schedule [Mesh] OR Shift Work Schedule [Text Word] OR Shift Work [Text Word] OR Work-Life Balance [Text Word] OR Work Life Conflict [Text Word] OR Work Family Balance [Text Word] OR Workload [Mesh] OR Workload [Text Word] OR Job Satisfaction [Mesh] OR Job Satisfaction [Text Word] OR Working Conditions [Mesh] OR Working Conditions [Text Word] OR Occupational Injuries [Mesh] OR Occupational Injuries [Text Word] OR Occupational Exposure [Mesh] OR Occupational Exposure [Text Word] OR organizational justice[Text Word] OR workplace justice [Text Word] OR decision authority [Text Word] OR job insecurity [Text Word] OR job seniority [Text Word] OR specialization [Text Word] OR work demands [Text Word] OR supervisor support [Text Word] OR low income [Text Word]
#3	Nurses [Mesh] OR Nurses [Text Word] OR Health Personnel [Mesh] OR Health Personnel [Text Word] OR Health Care Professional [Text Word] OR Students, Medical [Mesh] OR Students, Medical [Text Word] OR Medical Staff [Mesh] OR Medical Staff [Text Word] OR health professionals [Text Word] OR Physicians [Text Word]
Final	#1 AND #2 AND #3
Scopus	7,176
#1	"Burnout, Psychological" OR "Psychological Burnout" OR "Burnout Syndrome" OR "Burnout, Professional" OR "Occupational Burnout" OR "Career Burnout" OR "Emotional exhaustion" OR "physical exhaustion" OR "Job burnout" OR "Depersonalisation"
#2	"Job Strain" OR "Job demands" OR "job control" OR "work strain" OR "Occupational Stresses" OR "Occupational Stresses" OR "Job Stress" OR "Professional Stress" OR "Workplace Stress" OR "Work long hours" OR "Long working hours" OR "working hours" OR "Heavy overtime work" OR "Overtime work" OR "Occupational Factors" OR "Work Conditions" OR "Risk Factors" OR "Occupational Stress" OR "Workplace Bullying" OR "Job-related" OR "Occupational environment" OR "Professional environment" OR "Workplace Abuses" OR "Workplace" OR "effort reward imbalance" OR "Shift Work Schedule" OR "Shift Work" OR "Work-Life Balance" OR "Work Life Conflict" OR "Work Family Balance" OR "Workload" OR "Job Satisfaction" OR "Working Conditions" OR "Working Conditions" OR "Occupational Injuries" OR "Occupational Exposure" OR "organizational justice" OR "workplace justice" OR "decision authority" OR "job insecurity" OR "specialization" OR "work demands" OR "supervisor support" OR "low income"
#3	"Nurses" OR "Health Personnel" OR "Health Care Professional" OR "Students, Medical" OR "Medical Staff" OR "Health professionals" OR "Physicians"
Final	1 AND #2 AND #3
Web of Science	11,071
#1	TS=(Burnout, Psychological OR Psychological Burnout OR Burnout Syndrome OR Burnout, Professional OR Occupational Burnout OR Career Burnout OR Emotional exhaustion OR physical exhaustion OR Job burnout OR Depersonalisation)
#2	TS=(Job Strain OR Job demands OR job control OR work strain OR Occupational Stresses OR Occupational Stresses OR Job Stress OR Professional Stress OR Workplace Stress OR Work long hours OR Long working hours OR working hours OR Heavy overtime work OR Overtime work OR Occupational Factors OR Work Conditions OR Risk Factors OR Occupational Stress OR Workplace Bullying OR Job-related OR Occupational environment OR Professional environment OR Workplace

	Abuses OR Workplace OR effort reward imbalance OR Shift Work Schedule OR Shift Work OR Work-Life Balance OR Work Life Conflict OR Work Family Balance OR Workload OR Job Satisfaction OR Working Conditions OR Working Conditions OR Occupational Injuries OR Occupational Exposure OR organizational justice OR workplace justice OR decision authority OR job insecurity OR specialization OR work demands OR supervisor support OR low income)
#3	TS=(Nurses OR Health Personnel OR Health Care Professional OR Students, Medical OR Medical Staff OR Health professionals OR Physicians)
Final	1 AND #2 AND #3
