# Supplementary material for: Occupational Risk Factors for Burnout Syndrome Among Healthcare Professionals: A Global Systematic Review and Meta-Analysis
Source: Int J Environ Res Public Health. 2024 Nov 27;21(12):1583. doi: 10.3390/ijerph21121583 (PMC11675210; doi:10.3390/ijerph21121583)
Supplement: Supplementary file 1 [file ijerph-21-01583-s001.zip › Supplementary File S4.pdf]

Appendix S4: Characteristics of the studies included in the analyses

First author and year of publication	Country	Population	Study design	Age	Sex<br>% women	Sample size	Occupational risk factors	Burnout	Quality assessment				Results<br>r (N);<br>odd ratio and 95% confidence interval;<br>N, Mean, SD	Adjusted for
									Selection	Confounders	Data collection method	Withdrawals and dropouts		
Adler 2017	USA	Military Medical Personnel	Cross-sectional	≥18	41.2% women	344	1-Job demands	Maslach Burnout Inventory	Low	High	Low	Low	Emotional Exhaustion<br>0.07 (344)<br>Depersonalization<br>0.26 (344)	Unadjusted
Ahmed 2022	Ethiopia	health professionals	Cross-sectional	32±6.4	49.9% women	508	1-Working hours<br>>72 h<br>Reference ≤72 h	Maslach Burnout Inventory	Low	Moderate	Low	Low	Odd ratio<br>1.24 (CI 0.69-2.22)	Multivariate
Ajoudani 2019	Iran	Nurses	Cross-sectional	33.76 + 7.95	85.8% women	278	1-Workplace bullying	Maslach Burnout Inventory	Low	High	Low	Low	0.715 (278)	Unadjusted
Akkoç 2021	Turkey	Nurses	Cross-sectional	18-55	71.5% women	386	1-Workload<br>2-Job stress	Maslach Burnout Inventory	Moderate	High	Low	Low	Workload<br>0.52 (386)<br>Job stress<br>0.72 (386)	Unadjusted
Al-Dubai 2010	Yemen	Doctors	Cross-sectional	25-55	40.5% women	557	1-Working hours<br>>40 h<br>Reference ≤40 h	Maslach Burnout Inventory	Moderate	Moderate	Low	Low	Odd ratio<br>2.00 (CI 1.13-3.60)	Multivariate
Allen 2015	Australia	Nurses	Cross-sectional	46.5±11.5	89% women	672	1-Workplace bullying	Copenhagen Burnout Inventory	Low	High	Low	Low	0.38 (672)	Unadjusted
Alrawashdeh 2021	Jordan	Physicians	Cross-sectional	24-77	30.2% women	973	1-Working hours<br>>40 h<br>Reference ≤40 h	10-item Burnout Measure-Short version	Low	Moderate	Low	Low	Odd ratio<br>40-48 h<br>0.88 (CI 0.60-1.31)<br>>48 h<br>1.58 (CI 1.01-2.47)	Multivariate
Andlib 2022	Pakistan	Nurses	Cross-sectional	27.7 ± 4.4	76.7% women	288	1-Working hours<br>>48 h<br>Reference <48 h	Maslach Burnout Inventory	Low	High	Low	Low	emotional exhaustion<br>>48 h<br>73 (21.0 ± 11.1)<br><48 h<br>63 (25.0 ± 11.9)<br>depersonalization<br>>48 h<br>73 (9.7 ± 6.2)<br><48 h<br>63 (12.3 ± 6.3)	Unadjusted
Arigoni 2009	Switzerland	Physicians	Cross-sectional	Unknown	34% women	371	1-Working hours<br>>50 h<br>Reference ≤50 h	Maslach Burnout Inventory	Low	High	Low	Low	emotional exhaustion*<br>2.23 (CI 1.36-3.67)<br>depersonalization<br>2.33 (CI 1.36-3.97)	Unadjusted
Barello 2021	Italy	healthcare professionals	Cross-sectional	41.06±11.16	75% women	532	1-Job demands	Maslach Burnout Inventory	Moderate	Moderate	Low	Low	Odd ratio<br>2.35 (CI 1.67-3.32)	Adjusted (factors is unknown)

Belji Kangarlou 2022	Iran	Nurses	Cross-sectional	23-49	72.5% women	602	1-Job demands<br>2-1-Working hours<br>>12 h<br>Reference <8 h	Maslach Burnout Inventory	Moderate	High	Low	Low	Working hours<br>8 h<br>266 (85.4 ± 18.6)<br>12 h<br>90 (89.4 ± 16.9)<br>Mental demand<br>emotional exhaustion<br>0.74 (602)<br>depersonalization<br>0.51 (602)<br>Physical demand<br>emotional exhaustion<br>0.22 (602)<br>depersonalization<br>0.20 (602)<br>Temporal demand<br>emotional exhaustion<br>0.28 (602)<br>depersonalization<br>0.22 (602)	Unadjusted
Boo 2018	Malaysia	Doctors	Cross-sectional	28.5±3.7	61.7% women	313	1-Working hours	Maslach Burnout Inventory	Low	Moderate	Low	Low	Odd ratio<br>1.00 (CI 0.97-1.02)	Multivariate
Bourbonnais 1998	Canada	Nurses	Cross-sectional	23-65	Unknown	1,891	1-Job strain<br>2-Social support at work<br>3- job insecurity<br>4-Working hours<br>≥35 h<br>Reference <35 h	Maslach Burnout Inventory	Low	High	Low	Low	Prevalence ratio<br>Job strain<br>3.79 (CI 2.85–5.04)<br>Social support at work<br>1.83 (CI 1.60–2.09)<br>job insecurity<br>1.32 (CI 1.13–1.54)<br>Working hours<br>1.28 (CI 1.12–1.47)	Unadjusted
Cha 2022	Korea	Nurses	Cross-sectional	20-59	87.8% women	271	1-Job stress	A scale based on the Maslach Burnout Inventory	Low	High	Low	Low	0.499 (271)	Unadjusted
Chen 2020	China	Nurses	Cross-sectional	19-36	93.4% women	1,029	1-Job stress	Maslach Burnout Inventory	Low	High	Low	Low	Emotional exhaustion<br>0.465 (1029)<br>Depersonalization<br>0.366 (1029)	Unadjusted
Chen 2022	Taiwan	Nurses	Cross-sectional	39.93± 6.77	93.9% women	361	1-Working hours<br>≥51 h<br>Reference ≤40 h	Occupational Burnout Inventory	Low	High	Low	Low	≤ 40h<br>57 (35.08 ±16.38)<br>≥ 51h<br>81 (34.77 ± 17.03)	Unadjusted
Chowdhury 2022	Bangladesh	Nurses	Cross-sectional	28.41±5.54	49.27% women	1,264	1-Working hours<br>>48 h<br>Reference ≤36 h<br>2-workplace bullying<br>3-workplace violence	10-item Burnout Measure-Short version	Low	Moderate	Low	Low	Relative risk<br>Working hours<br>0.93 (CI 0.82-1.05)<br>1.22 (CI 1.04-1.42)<br>Workplace bullying<br>High risk<br>2.29 (CI 1.53–3.41)<br>Targeted<br>4.86 (CI 3.32–7.11)<br>Workplace violence<br>Low	Adjusted

													1.48 (CI 1.03–2.11)<br>Intermediate and high<br>3.65 (CI 2.40–5.56)	
Daryanto 2022	Indonesia	Health Profession als	Cross-sectional	Mean=34	65.6% women	1,077	1-Working hours<br>≥70h<br>Reference <70 h	Maslach Burnout Inventory	Low	High	Low	Low	Odd ratio<br>70-100 h<br>1.89 (CI 1.32-2.72)<br><70h<br>3.83 (CI 1.86 7.90)	Unknown
de Looff 2019	The Netherlands	Nurses	Longitudin al	21-59	59% women	110	1-job stress	Maslach Burnout Inventory	Moderat e	High	Low	Low	0.37 (110)	Unadjusted
de Wijn 2020	the Netherlands	Nurses	Cross-Sectional	42.4±11.3	75.6% women	692	1-job demands<br>2-Social support at work	Maslach Burnout Inventory	Low	High	Low	Low	job demands<br>0.49 (692)<br>social support supervisor<br>-0.22 (692)<br>Social support colleagues<br>-0.14 (692)	Unadjusted
Demirel Öğüt 2022	Turkey	Dermatolo gists	Cross-Sectional	Median=3 6	74.1% women	270	1-Working hours	Maslach Burnout Inventory	Low	High	Low	Low	Emotional exhaustion<br>0.121 (270)<br>Depersonalization<br>0.132 (270)	Unadjusted
Durand 2019	France	Health Profession als	Cross-sectional	37.7 ± 10.1	80.1% women	166	1-Job dissatisfaction	Maslach Burnout Inventory	Moderat e	Moderate	Low	Low	14.47 (CI 3.48-60.99)	Adjusted (factors are unknown)
Elay 2019	Turkey	Health Profession als	Cross-sectional	32.16±6.9 4	61% women	1,161	1-Working hours<br>≥80h<br>Reference <40 h	Maslach Burnout Inventory	Low	Moderate	Low	Low	Odd ratio**<br>emotional exhaustion<br>1.12 (CI 0.59-2.11)<br>depersonalization<br>1.47 (CI 0.80-2.68)	Multivariate
Escriba-Aguir 2006	Spain	doctors and nursing staff	Cross-sectional	Unspecifie d range	43.9% women	639	1-job demands<br>2-Job control<br>3-Social support at work<br>4-Workload	Maslach Burnout Inventory	Low	Moderate	Low	Low	Odd ratio<br>job demands<br>1.48 (CI 0.96–2.29)<br>Job control<br>Emotional exhaustion<br>1.65 (CI 1.04–2.63)<br>Depersonalization<br>1.15 (CI 0.77–1.73)<br>Supervisors' social support<br>Emotional exhaustion<br>1.64 (CI 1.04–2.59)<br>Depersonalization<br>1.25 (CI 0.84–1.85)<br>Co-workers' social support<br>Emotional exhaustion<br>1.54 (CI 0.91–2.61)<br>Depersonalization<br>1.00 (CI 0.63–1.62)<br>Dynamic physical workload<br>Emotional exhaustion<br>1.13 (CI 0.70–1.81)<br>Depersonalization<br>0.83 (CI 0.55–1.25)<br>Static physical workload	Multivariate

													Emotional exhaustion<br>1.33 (CI 0.79–2.27)<br>Depersonalization<br>1.15 (CI 0.74–1.79)	
Fu 2021	China	Nurses	Cross-sectional	Unspecified range	93.9% women	1,987	1-Violence at work	Maslach Burnout Inventory	Low	Low	Low	Low	Odd ratio**<br>Emotional exhaustion<br>2.65 (CI 1.78-3.96)<br>Cynicism<br>2.39 (CI 1.64-3.49)	"age, sex, marital status, educational level, monthly income, department, professional status, employment status, vacation days per year, work hours per week, number of child, and WPV experience"
Garcia-sierra 2016	Spain	Nurses	Cross-sectional	40.58±8.54	89.6% women	100	1-Job demands<br>2-Job control<br>3-Social support at work	Maslach Burnout Inventory	Moderate	High	Low	Low	Job demands<br>0.446 (100)<br>Job control<br>-0.112 (100)<br>Social support at work<br>-0.242 (100)	Unadjusted
Gherman 2022	Romania	Nurses	Cross-sectional	21-57	85.3% women	614	1-Job satisfaction	Maslach Burnout Inventory	Low	High	Low	Low	-0.42 (614)	Unadjusted
Gillet 2020	France	Nurses	Cross-sectional	23-64	90.2% women	378	1-Workload	Maslach Burnout Inventory	Low	High	Low	Low	0.478 (378)	Unadjusted
Goehring 2005	Switzerland	physicians	Cross-sectional	Unspecified range	16.4% women	1,755	1-Workload<br>2-Work-life imbalance	Maslach Burnout Inventory	Low	High	Low	Low	Odd ratio<br>Workload<br>Moderate burnout<br>1.70 (CI 1.30-2.10)<br>High burnout<br>2.20 (CI 1.30-5.70)<br>Work-life imbalance<br>Moderate burnout<br>1.8 (1.5- 2.3)<br>High burnout<br>2.2 (CI 1.2-4.1)	Unknown
Gouveia 2017	Brazil	medical residents	Cross-sectional	Unspecified range	51.9% women	129	1-Working hours	Maslach Burnout Inventory	Moderate	High	Low	Low	Emotional exhaustion<br>1.37 (CI 0.67-2.78)<br>Depersonalization<br>1.51 (CI 0.71-3.21)	Unadjusted
Guo 2021	China	Doctor<br>Nurses	Cross-sectional	Unspecified range	71.2% women	1,056	1-Working hours<br>>60 h<br>Reference <40 h	Maslach Burnout Inventory	Low	High	Low	Low	Working hours<br>Emotional exhaustion<br>< 40h<br>125 (19.52 ± 9.90)<br>> 60h	Unadjusted

							2-Violence at work						203 (28.01 ± 10.57)<br>Depersonalization<br>< 40h<br>125 (8.47 ± 5.92)<br>> 60h<br>203 (11.97 ± 5.94)<br>Violence at work<br>Emotional exhaustion<br>0.296 (1056)<br>Depersonalization<br>0.349 (1056)	
Ham 2022	Canada	Nurses	Cross-sectional	Unknown	70% women	611	1-Workload<br>2-work control	Maslach Burnout Inventory	Low	High	Low	Low	Workload<br>Emotional exhaustion<br>-0.525 (611)<br>Depersonalization<br>-0.187 (611)<br>Work control<br>Emotional exhaustion<br>-0.485 (611)<br>Depersonalization<br>-0.372 (611)	Unadjusted
Hamaideh 2011	Jordan	Nurses	Cross-sectional	21-54	44.2% women	181	1-Job satisfaction	Maslach Burnout Inventory	Low	High	Low	Low	Emotional exhaustion<br>-0.313 (181)<br>Depersonalization<br>-0.349 (181)	Unadjusted
Hausler 2018	Switzerland	Physicians	Cross-sectional	Unspecified range	Women	1,547	1-Effort-Reward Imbalance	Copenhagen Burnout Inventory	Low	High	Low	Low	High<br>356 (48.6 ±17)<br>Low<br>364 (32.2 ±14)	Unadjusted
Hayes 2015	Australia<br>New Zealand	Nurses	Cross-sectional	≥21	90.9% women	417	1-job stress<br>2-job satisfaction	Maslach Burnout Inventory	Low	High	Low	Low	job stress<br>Emotional exhaustion<br>0.52 (417)<br>Depersonalization<br>0.34 (417)<br>job satisfaction<br>Emotional exhaustion<br>-0.56 (417)<br>Depersonalization<br>-0.30 (417)	Unadjusted
Hong 2016	Korea	Nurses	Cross-sectional	21-49	Unknown	211	1-job stress	Maslach Burnout Inventory	Low	High	Low	Low	0.539 (211)	Unadjusted
Hu 2021	China	Doctor<br>Nurses	Cross-sectional	Unspecified range	68.7% women	2,411	1-Working hours<br>>40 h<br>Reference <40 h	Maslach Burnout Inventory	Low	Moderate	Low	Low	Odd ratio<br>41–50h<br>0.99 (CI 0.76-1.29)<br>51–60h<br>0.97 (CI 0.71-1.33)<br>>60h<br>1.12 (CI 0.75- 1.66)	Multivariate
Huo 2021	China	Doctor<br>Nurses<br>Medical technician	Cross-sectional	22-65	81.2% women	606	1-Working hours<br>>8 h<br>Reference <8	Maslach Burnout Inventory	Low	High	Low	Low	8-10h<br>1.26 (CI 0.89-1.78)<br>>10h<br>1.61 (CI 0.90-2.90)	Unadjusted
Ilhan 2008	Turkey	Nurses	Cross-sectional	30.6 ±5.4	Women	418	1-Working hours<br>>40 h<br>Reference ≤40	Maslach Burnout Inventory	Low	High	Low	low	Emotional exhaustion<br>≤40h<br>296 (17.46 ± 6.24)<br>>40h	Unadjusted

													122 (19.28 ± 6.44)<br>Depersonalization<br>≤40h<br>296 (5.36 ± 3.92)<br>>40h<br>122 (6.60 ± 3.60)	
Janssen 1999	the Netherlands	Nurses	Cross-sectional	34±8.71	91% women	156	1-Workload<br>2-social support at work	Maslach Burnout Inventory	Low	High	Low	Moderate	Workload<br>Emotional exhaustion<br>0.45 (156)<br>Depersonalization<br>0.07 (156)<br>Support of co-workers<br>Emotional exhaustion<br>-0.31 (156)<br>Depersonalization<br>-0.22 (156)<br>Support of supervisor<br>Emotional exhaustion<br>-0.33 (156)<br>Depersonalization<br>-0.22 (156)	Unadjusted
Jonge 1996	The Netherlands	Nurses	Cross-sectional	18-58	82% women	220	1-Job demands<br>2-social support at work	Maslach Burnout Inventory	Moderat e	High	Low	Low	Job demands<br>0.49 (220)<br>social support at work<br>-0.15 (220)	Unadjusted
Karakoc 2016	Turkey	Nurses	Cross-sectional	≥20	93.5% women	171	1-Working hours<br>>50 h<br>Reference ≤40	Maslach Burnout Inventory	Low	High	Low	Low	Emotional exhaustion<br>40h<br>59 (12.61 ± 6.67)<br>>50h<br>12 (15.58 ± 7.06)<br>Depersonalization<br>40h<br>59 (4.03 ± 3.63)<br>>50h<br>12 (5.67 ± 2.81)	Unadjusted
Khamisa 2016	South Africa	Nurses	Longitudin al	≥20	Unknown	277	1- Job demands	Maslach Burnout Inventory	Low	High	Low	Low	Odd ratio<br>1.80 (CI 0.97–3.32)	Unknown
Khan 2022	Belgium	Nurses	Cross-sectional	≥18	84% women	4,552	1-Workload	Maslach Burnout Inventory	Low	Moderate	Low	Low	Odd ratio<br>2.1 (CI 1.5–2.8)	Multivariate
Kim 2019	Korea	Nurses	Cross-sectional	Unknown	96.3% women	324	1-Workplac bullying	Maslach Burnout Inventory	Low	High	Low	Low	Emotional exhaustion<br>0.554 (324)<br>Depersonalization<br>0.470 (324)	Unadjusted
Kushnir 2000	Israel	Physicians	Cross-sectional	47.1+8.3<br>Pediatricia ns<br>43.1±6.9	41.4% women	309	1-job satisfaction<br>2-job stress	Burnout in work organizati ons	Low	High	Low	Low	job satisfaction<br>-0.44 (309)<br>job stress<br>0.50 (309)	Unadjusted
Labrague 2020	Philippines	Nurses	Cross-sectional	29.80±<br>7.80	78.7% women	549	1-job satisfaction<br>2-Job stress	Copenhag en Burnout Inventory	Low	High	Low	Low	job satisfaction<br>-0.20 (549)<br>Job stress<br>0.17 (549)	Unadjusted
Lee 2020 (1)	Canada	Nurses	Cross-sectional	48.7 ±12.1	99.1% women	113	1-Workload<br>2-job satisfaction	Maslach Burnout Inventory	Moderat e	High	Low	Low	Workload<br>0.57 (113)<br>job satisfaction<br>-0.63 (113)	Unadjusted

Lee 2020 (2)	Korea	Oncologists	Cross-sectional	32-63	44.1% women	111	1-Working hours 60-80h Reference ≤60	Maslach Burnout Inventory	Moderate	Moderate	Low	Low	Odd ratio*** 0.87 (CI 0.67-1.06)	Multivariate
Lee 2022	Korea	Nurses	Cross-sectional	Unspecified range	86% women	146	1-job stress	Maslach Burnout Inventory	Low	High	Low	Low	0.13 (146)	Unadjusted
Leineweber 2014	Sweden	Nurses	Cross-sectional	22-67	93.4% women	8,620	1-work-family conflict	Maslach Burnout Inventory	Low	Low	Low	Low	Odd ratio emotional exhaustion 1.23 (CI 1.08-1.40) Depersonalization 1.11 (CI 0.89-1.38)	age, sex, baccalaureate degree in nursing, years of experience as RN, work-family conflict, department level variables,
Leiter 2005	Canada	Nurses	Cross-sectional	Unknown	95.7% women	545	1- Workload 2-job control	Maslach Burnout Inventory	Low	High	Low	Low	Workload -0.714 (545) job control -0.316 (545)	Unadjusted
Li 2018	China	Anesthesiologists	Cross-sectional	Unspecified range	55% women	2,873	1-Working hours ≥40h Reference <40	Maslach Burnout Inventory	Low	Moderate	Low	Low	Odd ratio 40–49h 0.90 (CI 0.58–1.40) 50–59h 1.32 (CI 0.84–2.06) 60-69h 1.72 (CI 1.05–2.84) ≥70h 2.38 (CI 1.28–4.43)	Multivariate
Li 2022	China	Nurses	Cross-sectional	22-55	92.9% women	509	1- Job strain	Maslach Burnout Inventory	Low	High	Low	Low	Emotional exhaustion 0.514 (509) Depersonalization 0.361 (509)	Unadjusted
Liao 2022	China	Nurses	Cross-sectional	30.16±7.62	Unknown	488	1-job stress	Maslach Burnout Inventory	Low	High	Low	Low	Emotional exhaustion 0.48 (488) Depersonalization 0.36 (488)	Unadjusted
Liu 2018	China	Nurses	Cross-sectional	Unspecified range	96.6% women	1,761	1-job satisfaction 2--Violence at work	Maslach Burnout Inventory	Low	High	Low	Moderate	job satisfaction -0.562 (1761) Violence at work 0.206 (1761)	Unadjusted
Liu 2020	China	Doctors Nurses Medical technicians	Cross-sectional	34.06± 9.22	79.08% women	1,052	1-Workload	Maslach Burnout Inventory	Low	Moderate	Low	Low	Odd ratio Moderate Burnout 1.09 (CI 1.06-1.13) Serious Burnout 1.34 (CI 1.22-1.47)	sex, professional group, department, hospital level
Medeiros 2022	Brazil	Health Professionals	Cross-sectional	29-38 interquartile range	67% women	265	1-Workload	Maslach Burnout Inventory	Low	High	Low	Low	Odd ratio Emotional exhaustion 1.89 (CI 1.04-3.58) Depersonalization 2.37 (CI 2.02-5.50)	Unadjusted
Meng 2021	China	Nurses	Cross-sectional	31.53± 6.97	80.8% women	198	1-Working hours	Maslach Burnout Inventory	Low	High	Low	Low	Odd ratio 1.74 (CI 1.07-2.83)	Unknown

Mijakoski 2015	Macedonian	Nurses Physicians	Cross-sectional	Unknown	49% women in physicians 93% women in nurses	286	1- Job demands	Maslach Burnout Inventory	Low	High	Low	Low	physicians<br>Emotional exhaustion<br>Physical demands<br>0.229 (138)<br>Organizational demands<br>0.104 (138)<br>Emotional demands<br>0.248 (138)<br>Depersonalization<br>Physical demands<br>0.088 (138)<br>Organizational demands<br>0.126 (138)<br>Emotional demands<br>0.221 (138)<br>Nurses<br>Emotional exhaustion<br>Physical demands<br>0.336 (148)<br>Organizational demands<br>0.387 (148)<br>Depersonalization<br>Physical demands<br>0.200 (148)<br>Organizational demands<br>0.272 (148)	Unadjusted
Min 2022	Korea	Nurses	Cross-sectional	25-67	Unknown	155	1-Work-life balance	Maslach Burnout Inventory	Low	High	Low	Low	-0.45 (155)	Unadjusted
Molero Jurado 2022	Spain	Nurses	Cross-sectional	34.71 ±9.35	88.05% women	1,013	1- Job control<br>2-Social support at work<br>3-Demands	Maslach Burnout Inventory	Low	High	Low	Low	Job control<br>Emotional exhaustion<br>-0.193 (1013)<br>Depersonalization<br>-0.184 (1013)<br>Social support at work<br>Emotional exhaustion<br>-0.285 (1013)<br>Depersonalization<br>-0.181 (1013)<br>Demands<br>Emotional exhaustion<br>0.397 (1013)<br>Depersonalization<br>0.255 (1013)	Unadjusted
Montgomery 2006	Greece	Doctors	Cross-sectional	25-62	44% women	162	1- Job demands<br>2-Working hours<br>3-Work-family interference	Maslach Burnout Inventory	Low	High	Low	Low	Emotional exhaustion<br>Quantitative job demands<br>0.36 (162)<br>Emotional job demands<br>0.43 (162)<br>Depersonalization<br>Quantitative job demands<br>0.22 (162)<br>Emotional job demands<br>0.24 (162)<br>Working hours<br>Emotional exhaustion<br>0.17 (162)<br>Depersonalization	Unadjusted

												0.27 (162)<br>Work-family interference<br>Emotional exhaustion<br>0.62 (162)<br>Depersonalization<br>0.38 (162)	
Montgomery 2015	seven European countries	Nurses	Cross-sectional	45.75±9.95	92.8% women	1,279	1-Workload<br>2- Job demands	Maslach Burnout Inventory	Low	High	Low	Low	Workload<br>Emotional exhaustion<br>0.363 (1279)<br>Depersonalization<br>0.248 (1279)<br>Organizational demands<br>Emotional exhaustion<br>0.420 (1279)<br>Depersonalization<br>0.390 (1279)<br>Emotional demands<br>Emotional exhaustion<br>0.428 (1279)<br>Depersonalization<br>0.423 (1279)	Unadjusted
Moreira 2020	Brazil	mental health professionals	Cross-sectional	Unspecified range	64.8% women	293	1-job satisfaction<br>2- Job demands<br>3-job control<br>4-Social support at work	Maslach Burnout Inventory	Low	High	Low	Low	Odd ratio<br>job satisfaction<br>5.00 (CI 1.46-17.09)<br>Job demands<br>Emotional exhaustion<br>10.22 (CI 4.64-22.52)<br>Depersonalization<br>3.24 (CI 1.84-5.70)<br>job control<br>Emotional exhaustion<br>2.01 (CI 1.04- 3.14)<br>Depersonalization<br>1.14 (CI 0.66-1.99)<br>Social support at work<br>Emotional exhaustion<br>4.43 (CI 2.24- 8.76)<br>Depersonalization<br>1.41 (CI 0.82-2.43)	Unknown
Nishimura 2014	Japan	Physicians	Cross-Sectional	21.7±9.1	Both % women unspecified	2,635	1-Working hours	Maslach Burnout Inventory	Low	Moderate	Low	Low	Odd ratio<br>1.11 (CI 1.02–1.21)	Multivariate
Ntantana 2017	Greece	Nurses Physicians	Cross-Sectional	≥20	25.8% women	469	1-job satisfaction	Maslach Burnout Inventory	Low	Moderate	Low	Low	Odd ratio<br>0.25 (CI 0.13-0.47)	Multivariate
Ozkula 2017	Turkey	Physicians	Cross-Sectional	≥20	58.5% women	258	1-Working hours<br>>8h<br>Reference ≤40	Maslach Burnout Inventory	Low	High	Low	Low	Emotional exhaustion<br>≤8 h<br>40 (20.32± 6.52)<br>>8 h<br>218 (24.07±7.69)<br>Depersonalization<br>≤8 h<br>40 (12.85± 3.30)<br>>8 h<br>218 (14.29± 4.24)	Unadjusted

Ozyurt 2006	Turkey	Physicians	Cross-Sectional	Unspecified range	36% women	598	1-job satisfaction	Maslach Burnout Inventory	Low	High	Low	Low	Emotional exhaustion -0.559 (598) Depersonalization -0.368 (598)	Unadjusted
Page 2021	Australia	Nurses	Cross-Sectional	26-65	93% women	153	1-Job control 2-Job demands	a single-item measure of burnout	Moderate	High	High	Low	Job control -0.25 (153) Job demands 0.28 (153)	Unadjusted
Pang 2021	China	physicians nurses	Cross-Sectional	Unspecified range	80.4% women	862	1-working hours	Maslach Burnout Inventory	Low	High	Low	Low	Odd ratio 2.93 (CI 1.16-7.45)	
Park 2015	Korea	Nurses	Cross-Sectional	≥22	Both % women unspecified	419	1-job satisfaction 2-1-job stress	Unspecified	Low	High	High	Low	job satisfaction 0.14 (419) job stress 0.63 (419)	Unadjusted
Pedrini 2009	Italy	mental health professionals	Cross-Sectional	≥18	64% women	202	1-Workload	Maslach Burnout Inventory	Low	Moderate	Low	Low	Odd ratio** 1.36 (CI 0.35-5.32)	Multivariate
Peng 2022	China	Nurses	Cross-Sectional	30.61±7.45	93.5% women	493	1-workplace Bulling	Professional Quality of Life scale	Low	High	Low	Low	0.458 (493)	Unadjusted
Pinho 2022	Brazil	Residents	Cross-Sectional	27.8±4.4	78.1% women	1,313	1-working hours >60 h	Oldenburg Burnout Inventory	Low	Moderate	Low	Low	Odd ratio 1.36 (CI 1.03-1.79)	Multivariate
Pu 2017	China	Neurologist	Cross-Sectional	≥20	Men	5,558	1-Working hours >45h Reference <45 2-job stress 3-job satisfaction	Maslach Burnout Inventory	Low	Moderate	Low	Low	Odd ratio 45-55h 1.18 (CI 0.90-1.54) >55 1.41 (CI 1.08–1.84) Job stress 2.96 (CI 2.44–3.59) job satisfaction 2.30 (CI 1.85–2.87)	Multivariate
Qiao 2016	China	physicians nurses	Cross-Sectional	19-60	70.1% women	492	1-job satisfaction	Maslach Burnout Inventory	Low	High	Low	Low	Odd ratio 2.83 (CI 1.52-5.29)	Unknown
Rabatin 2016	USA	family physicians and general internists	Cross-Sectional	Unknown	44.3% women	422	1-jon control	Maslach Burnout Inventory	Low	High	Low	Low	Odd ratio 0.12 (CI 0.04-0.30)	Unknown
Rasmussen 2016	Australia	psychosocial oncologists	Cross-Sectional	20-69	84.2% women	417	1-Effort–reward imbalance	Maslach Burnout Inventory	Low	High	Low	Low	Odd ratio 1.03 (CI 0.97-1.08)	Unknown
Rostamabadi 2019	Iran	Nurses	Cross-Sectional	19-62	78.4% women	522	1-Job demands 2-Social support at work	Maslach Burnout Inventory	Low	High	Low	Low	Psychological job demands Emotional exhaustion 0.058 (522) Depersonalization 0.236 (522) Physical job demands Emotional exhaustion 0.163 (522) Depersonalization	Unadjusted

													0.152 (522)<br>Social support at work Emotional exhaustion<br>-0.171 (522)<br>Depersonalization<br>-0.009 (522)	
See 2018	Asian countries	physicians nurses	Cross-Sectional	Unknown	Both % women unspecified	4,092	1-Work‑life balance<br>2-Working hours	Maslach Burnout Inventory	Low	Moderate	Low	Low	Odd ratio<br>Physicians<br>Work‑life balance<br>0.92 (CI 0.81–1.04)<br>Working hours<br>1.02 (CI 0.98–1.07)<br>Nurses<br>Work‑life balance<br>0.87 (CI 0.81–0.95)<br>Working hours<br>0.96 (CI 0.93–1.00)	Multivariate
Shanafelt 2022	USA	Physicians	Cross-Sectional	IQR 41-60	48.3% women	2,440	1-Working hours	Maslach Burnout Inventory	Low	Moderate	Low	Moderate	Odd ratio<br>1.02 (1.01-1.03)	Multivariate
Silva 2015	Brazil	Nurses	Cross-sectional	28-42	50% women	130	1-Job strain	Maslach Burnout Inventory	Moderate	Moderate	Low	Low	Odd ratio<br>0.69 (CI 0.25-1.89)	Adjusted
Smith 2023	Australia	Nurses	Cross-Sectional	20–76	85.9% women	383	1-job satisfaction	Maslach Burnout Inventory	Low	High	Low	Low	Emotional exhaustion<br>-0.58 (383)<br>Depersonalization<br>-0.34 (383)	Unadjusted
Sugawara 2017	Japan	Nurses	Cross-Sectional	48.6±11.5	77.2% women	180	1- Work–family conflict<br>2- workload<br>3-job demands	Maslach Burnout Inventory	Low	High	Low	Low	Work–family conflict<br>emotional exhaustion<br>0.707 (180)<br>Cynicism<br>0.585 (180)<br>Workload<br>emotional exhaustion<br>0.524 (180)<br>Cynicism<br>0.307 (180)<br>Job demands<br>emotional exhaustion<br>0.372 (180)<br>Cynicism<br>0.159 (180)	Unadjusted
Sundin 2011	Sweden	Nurses	Longitudinal	23–64	94.3% women	580	1-Social support at work<br>2-job demands	Maslach Burnout Inventory	Low	Moderate	Low	Low	Odd ratio<br>job demands<br>1.82 (CI 0.96–3.44)<br>Social support at work<br>2.58 (CI 1.24–5.37)	Multivariate
Surgenor 2013	New Zealand	medical consultants	Cross-Sectional	48 ±7.7	27% women	267	1-Working hours<br>2-job satisfaction	Maslach Burnout Inventory	Low	High	Low	Low	Odd ratio<br>Working hours<br>1.04 (CI 1.00-1.06)<br>job satisfaction<br>0.372 (CI 0.220-0.628)	Unadjusted
Tang 2018	China	oncology professionals	Cross-Sectional	≥21	80.4% women	862	1-effort-reward imbalance	Maslach Burnout Inventory	Low	High	Low	Low	Odd ratio<br>Emotional exhaustion<br>103.67 (CI 12.21-879.89)<br>Depersonalization<br>52.00 (CI 8.23-328.85)	Unknown

Tavakoli 2018	Iran	Nurses	Cross-Sectional	23-54	58.9% women	709	1-job satisfaction<br>2-job stress	Maslach Burnout Inventory	Low	High	Low	Low	job satisfaction<br>-0.41 (709)<br>job stress<br>0.57 (709)	Unadjusted
Teixeira 2013	Portugal	doctors<br>nurses	Prospective	27-38	65% women	267	1-Working hours<br>>40h<br>Reference =35h	Maslach Burnout Inventory	Moderate	Moderate	Low	Low	Odd ratio<br>40 hours<br>0.733 (CI 0.257-2.089)<br>42 hours<br>1.340 (CI 0.341-5.269)	Multivariate
Tremolada 2015	Italy	physicians<br>nurses	Cross-Sectional	48.38± 9.22	73.4% women	470	1-job satisfaction<br>2-job stress	Link Burnout Questionnaire	Moderate	High	Moderate	Low	job satisfaction<br>physical exhaustion<br>0.42 (200)<br>relationship with patient<br>0.22 (469)<br>Professional inefficacy<br>0.25 (469)<br>Burnout Disillusion<br>0.44 (469)<br>job stress<br>Physicians<br>-0.22 (219)<br>Nurses<br>-0.22 (270)	Unadjusted
Tsou 2021	Taiwan	Nurses	Cross-Sectional	35.20 ±11.02	95.8% women	1,758	1-Working hours<br>>45h<br>Reference ≤45h	Maslach Burnout Inventory	Low	Moderate	Low	Low	Odd ratio<br>46-50h<br>0.94 (CI 0.64-1.56)<br>51-59 h<br>1.35 (CI 1.02-2.11)<br>≧60 h<br>0.72 (CI 0.13-2.56)	Multivariate
Udho 2022	Uganda	Nurses	Cross-Sectional	Unspecified range	56.5% women	395	1-Working hours<br>>10h<br>Reference <10h	Professional Quality of Life	Low	Moderate	Low	Low	Odd ratio**<br>3.22 (CI 1.46-7.10)	Adjusted ((factors are unknown))
Van Bogaert 2013	Belgium	Nurses	Cross-Sectional	38.3± 10.3	85.2% women	1,201	1-Workload	Maslach Burnout Inventory	Moderate	High	Low	Low	Emotional exhaustion<br>0.516 (1201)<br>Depersonalization<br>0.231 (1201)	Unadjusted
Villarreal-Zegarra 2022	Peru	Physicians	Cross-Sectional	23-65	31.2% women	2,100	1-job satisfaction	Maslach Burnout Inventory	Low	High	Low	Low	Emotional exhaustion<br>-0.443 (2100)<br>-0.300 (2100)<br>-0.460 (2100)<br>-0.199 (2100)<br>Depersonalization<br>-0.382 (2100)<br>-0.261 (2100)<br>-0.348 (2100)<br>-0.187 (2100)	Unadjusted
Viotti 2016	Italy	Nurses	Cross-Sectional	22-62	82.2% women	522	1-job satisfaction<br>2-job demands	Maslach Burnout Inventory	Low	High	Low	Low	job satisfaction<br>-0.52 (522)<br>job demands<br>0.33 (522)	Unadjusted
Wang 2012	China	Nurses	Cross-Sectional	34.53 ±8.55	Women	1,332	1-Work family conflict	Maslach Burnout Inventory	Low	High	Low	Low	Emotional Exhaustion<br>0.481 (1332)<br>Cynicism<br>0.342 (1332)	Unadjusted

Wang 2014	China	Physicians	Cross-Sectional	39.13± 9.62	59.5% women	457	1-Working hours<br>>40h<br>Reference ≤40h<br>2-job demands<br>3-job control<br>4-social support at work<br>5-effort-reward imbalance	Maslach Burnout Inventory	Low	Moderate	Low	Low	Odd ratio<br>40−60h<br>3.63 (CI 2.32−5.69)<br>>60h<br>4.54 (CI 2.04−10.13)<br>job demands<br>1.28 (CI 0.78−2.11)<br>2.98 (CI 1.75−5.07)<br>Job control<br>1.16 (CI 0.70−1.91)<br>1.64 (CI 0.95−2.83)<br>social support at work<br>1.42 (CI 0.88−2.29)<br>2.62 (CI 1.50−4.59)<br>effort-reward imbalance<br>9.19 (CI 4.15−20.39)	Multivariate
Wang 2019	China	Nurses	Cross-Sectional	19-57	93.4% women	2,504	1-Home-work interface<br>2-job satisfaction<br>3-job control<br>4-job stress	Maslach Burnout Inventory	Low	High	Low	Low	Home-work interface<br>-0.51 (2504)<br>job control<br>-0.43 (2504)<br>Job stress<br>-0.49 (2504)<br>Job satisfaction<br>-0.53 (2504)	Unadjusted
Wu 2013	China	Doctors	Cross-Sectional	38.67 ±8.78	53.8% women	1,202	1-Working hours<br>>40h<br>Reference ≤40h<br>2-job demands<br>3-Social support at work	Maslach Burnout Inventory	Low	High	Low	Low	Working hours<br>Emotional exhaustion<br>≤40h<br>458 (9.70 ± 6.75)<br>>40h<br>744 (11.94 ± 7.64)<br>Cynicism<br>≤40h<br>458 (5.77 ± 4.61)<br>>40h<br>744 (7.25 ± 5.26)<br>job demands<br>Emotional exhaustion<br>0.39 (1202)<br>Cynicism<br>0.21 (1202)<br>Supervisor support<br>Emotional exhaustion<br>-0.22 (1202)<br>Cynicism<br>-0.24 (1202)<br>Coworker support<br>Emotional exhaustion<br>-0.10 (1202)<br>Cynicism<br>-0.15 (1202)	Unadjusted
Xian 2020	China	Nurses	Cross-Sectional	≥20	Men	366	1-job control<br>2-Workload<br>3-Social support at work	Maslach Burnout Inventory	Low	High	Low	Low	job control<br>Emotional exhaustion<br>0.004 (366)<br>Cynicism<br>0.017 (366)<br>Workload<br>Emotional exhaustion<br>0.618 (366)	Unadjusted

													Cynicism<br>0.349 (366)<br>Social support at work<br>Emotional exhaustion<br>0.439 (366)<br>Cynicism<br>0.333 (366)	
Xie 2011	China	Nurses	Cross-Sectional	Mean= 32.11	Women	527	1-job demands<br>2- Job control<br>3-effort-reward imbalance<br>4-Social support at work	Maslach Burnout Inventory	Low	Moderate	Low	Low	Odd ratio<br>job demands<br>Emotional exhaustion<br>1.85 (CI 1.06–3.23)<br>4.99 (CI 3.01–8.26)<br>Depersonalization<br>1.52 (CI 0.86–2.69)<br>3.15 (CI 1.88–5.29)<br>Job control<br>Emotional exhaustion<br>1.21 (0.76–1.94)<br>2.09 (1.28–3.38)<br>Depersonalization<br>0.95 (0.58–1.56)<br>1.28 (0.77–2.14)<br>effort-reward imbalance<br>Emotional exhaustion<br>5.51 (3.63–8.36)<br>Depersonalization<br>3.18 (2.12–4.76)<br>Social support at work<br>Emotional exhaustion<br>1.10 (0.68–1.77)<br>2.13 (1.33–3.41)<br>Depersonalization<br>1.30 (0.77–2.18)<br>2.40 (1.44–3.99)	Multivariate
Yang 2017	China	physician's nurses public health workers	Cross-Sectional	Unspecified range	59.6% women	1,382	1- Work-family conflict<br>2-Job satisfaction	Unknown	Low	High	High	Low	Work-family conflict<br>0.063 (1382)<br>0.111 (1382)<br>0.155 (1382)<br>Job satisfaction<br>-0.479 (1382)	Unadjusted
Yao 2021	China	Psychiatrists	Cross-Sectional	30-39	58.1% women	4,520	1-Working hours<br>>40h<br>Reference ≤40h	Maslach Burnout Inventory	Low	High	Low	Low	Odd ratio<br>41–50h<br>1.47 (CI 1.08–2.01)<br>51–60h<br>2.39 (CI 1.71–3.34)<br>≥ 61<br>3.09 (CI 2.25–4.25)	Unknown
Yeh 2021	Taiwan	Nurses	Cross-Sectional	20≥	92.9% women	239	1-Social support at work<br>2-work–family conflict	Maslach Burnout Inventory	Low	High	Low	Low	Social support at work<br>Emotional exhaustion<br>−0.189 (239)<br>Depersonalization<br>−0.212 (239)<br>Work–family conflict<br>Emotional exhaustion<br>0.710 (239)<br>Depersonalization<br>0.562 (239)	Unadjusted

Yoon 2016	Korea	Nurses	Cross-Sectional	20-50	Women	236	1-Job satisfaction	Maslach Burnout Inventory	Low	High	Low	Low	-0.435 (236)	Unadjusted
Yu 2019	China	Neurosurgeons	Cross-Sectional	Unspecified range	7.07% women	1,202	1-Working hours >55h	Maslach Burnout Inventory	Low	Moderate	Low	Low	Odd ratio<br>56–70h<br>0.63 (CI 0.49-0.81)<br>≥71h<br>3.12 (CI 2.43-3.99)	Multivariate
Yu 2020	China	orthopedic surgeon neurosurgeon	Cross-Sectional	29.21± 6.82	93.4% women	1,333	1-Working hours >55h	Maslach Burnout Inventory	Low	Moderate	Low	Low	Odd ratio<br>56–70h<br>1.13 (CI 0.82–1.56)<br>0.55 (CI 0.39–0.77)<br>≥71h<br>2.24 (CI 1.58–3.18)<br>3.61 (CI 2.60–5.01)	Multivariate
Žutautiene 2020	Lithuania	Physicians	Cross-Sectional	39.7±13.58	65.7% women	647	1-job demands<br>2-job control<br>3-Job insecurity	Copenhagen Burnout Inventory	Low	Moderate	Low	Low	Odd ratio<br>job control<br>0.264 (CI 0.137-0.511)<br>job demands<br>3.48 (CI 1.92-6.29)<br>Job insecurity<br>1.50 (CI 0.97–2.31)	Multivariate

*Calculated by author(s) **Reversed score calculated by author(s) ***Confidence interval score calculated by author(s
