# Supplementary material for: Occupational Risk Factors for Burnout Syndrome Among Healthcare Professionals: A Global Systematic Review and Meta-Analysis
Source: Int J Environ Res Public Health. 2024 Nov 27;21(12):1583. doi: 10.3390/ijerph21121583 (PMC11675210; doi:10.3390/ijerph21121583)
Supplement: Supplementary file 1 [file ijerph-21-01583-s001.zip › Supplementary File S5.pdf]

Appendix S5: Quality assessment framework

Type of bias	Criteria definition	Classification (potential for bias)
Selection bias	Sampling method of the study population, representativeness (response rate, difference between responders and non-responders, investigate and control of variables in case of difference between responders and non-responders)	**Low:** Target population defined as representative of the general population or subgroup of the general population (specific age group, women, men, specific geographic area, and specific occupational group) and response rate is 80% or more.<br>**Moderate:** Target population defined as somewhat representative of the general population, a restricted subgroup of the general population, response rate 60%-79%.<br>**High:** Target population defined as "self-referred"/ volunteers, response rate less than 60%.
Confounders	Matching two groups<br>Stratification<br>Statistical analysis	**Low:** Controlled for most potential confounding factors including age and sex.<br>**Moderate:** Controlled for few potential confounding factors, including both age and sex.<br>**High:** Not controlled for both age and sex, or controlled for less than two confounding factors.
Data collection method	Clear definition of outcome Standard method for outcome assessment Outcome assessors blinded to exposure status	**Low:** Valid and reliable tools for data collection.<br>**Moderate:** Valid and not reliable tools or reliability is not described.<br>**High:** Without validity and reliability or both reliability and validity are not described.
Withdrawals and drop-outs	Withdrawals and drop-out rates<br>Size of missing data	**Low:** Follow up participation rate of 80% or higher or missing data on less than 20%.<br>**Moderate:** Follow up participation rate of 60%-79%, or missing data on 20%-40%.<br>**High:** Follow up participation rate of less than 60%, or missing data on more than 40%.
