# Supplementary figures and images for: Occupational Risk Factors for Burnout Syndrome Among Healthcare Professionals: A Global Systematic Review and Meta-Analysis
Source: Int J Environ Res Public Health. 2024 Nov 27;21(12):1583. doi: 10.3390/ijerph21121583 (PMC11675210; doi:10.3390/ijerph21121583)

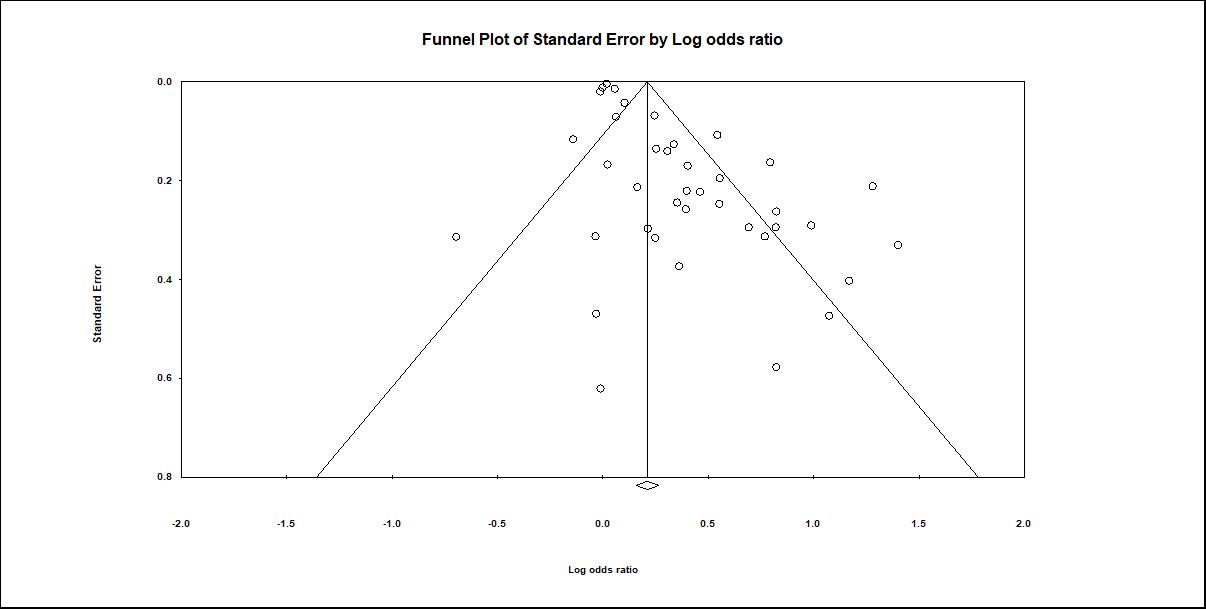

Supplement: Supplementary file 1 [file ijerph-21-01583-s001.zip › Figure S1.tiff]

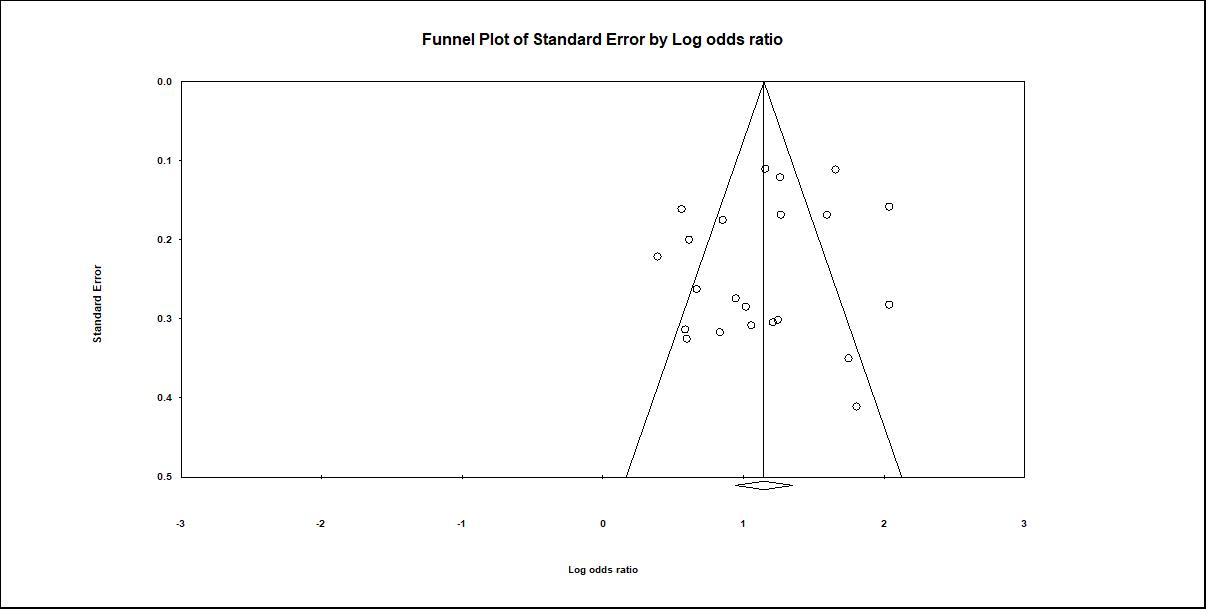

Supplement: Supplementary file 1 [file ijerph-21-01583-s001.zip › Figure S2.tiff]

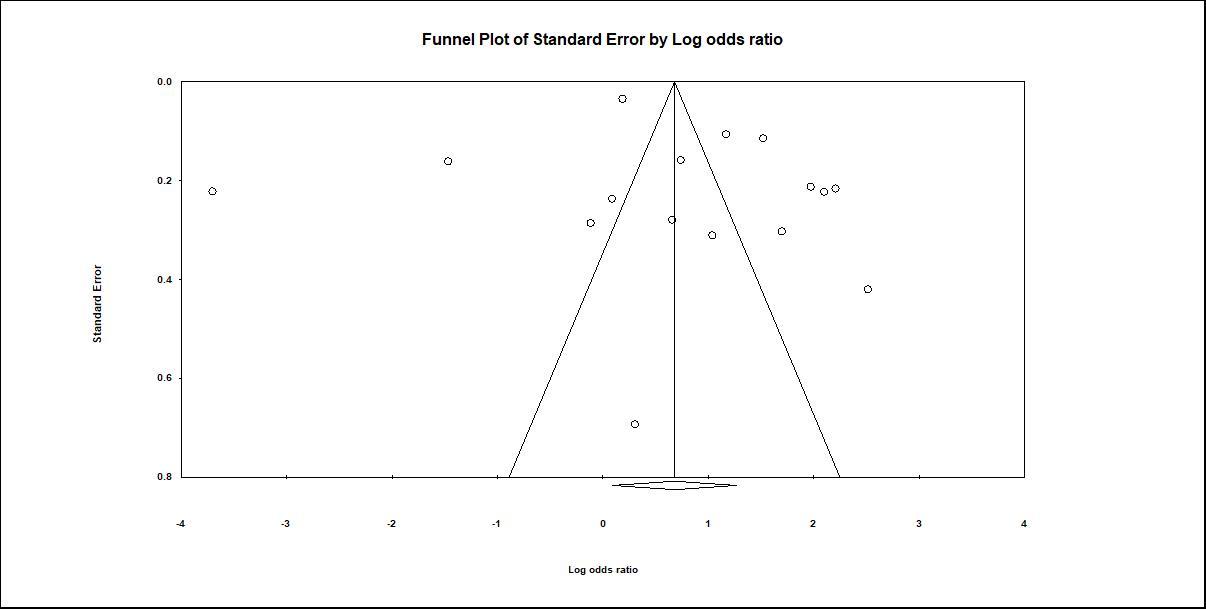

Supplement: Supplementary file 1 [file ijerph-21-01583-s001.zip › Figure S3.tiff]

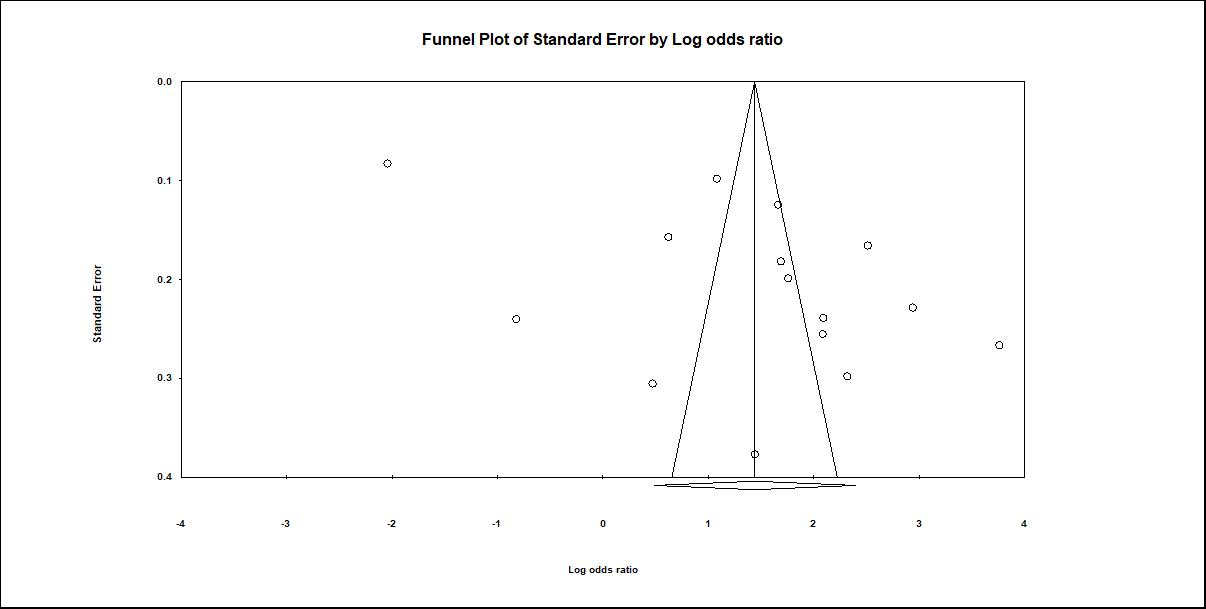

Supplement: Supplementary file 1 [file ijerph-21-01583-s001.zip › Figure S4.tiff]

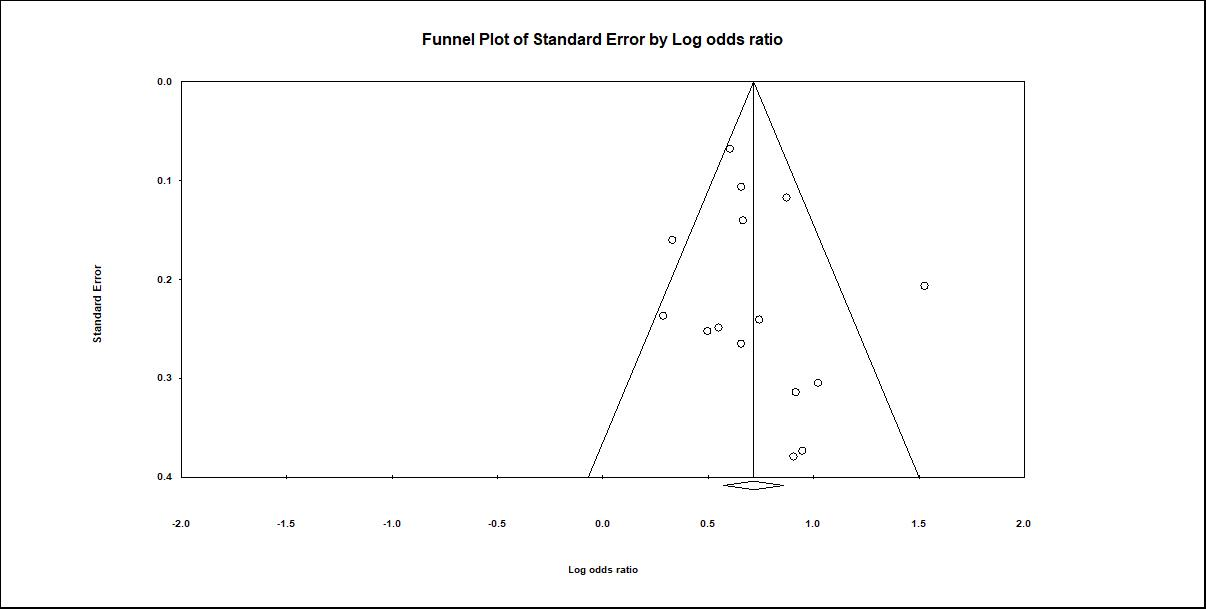

Supplement: Supplementary file 1 [file ijerph-21-01583-s001.zip › Figure S5.tiff]

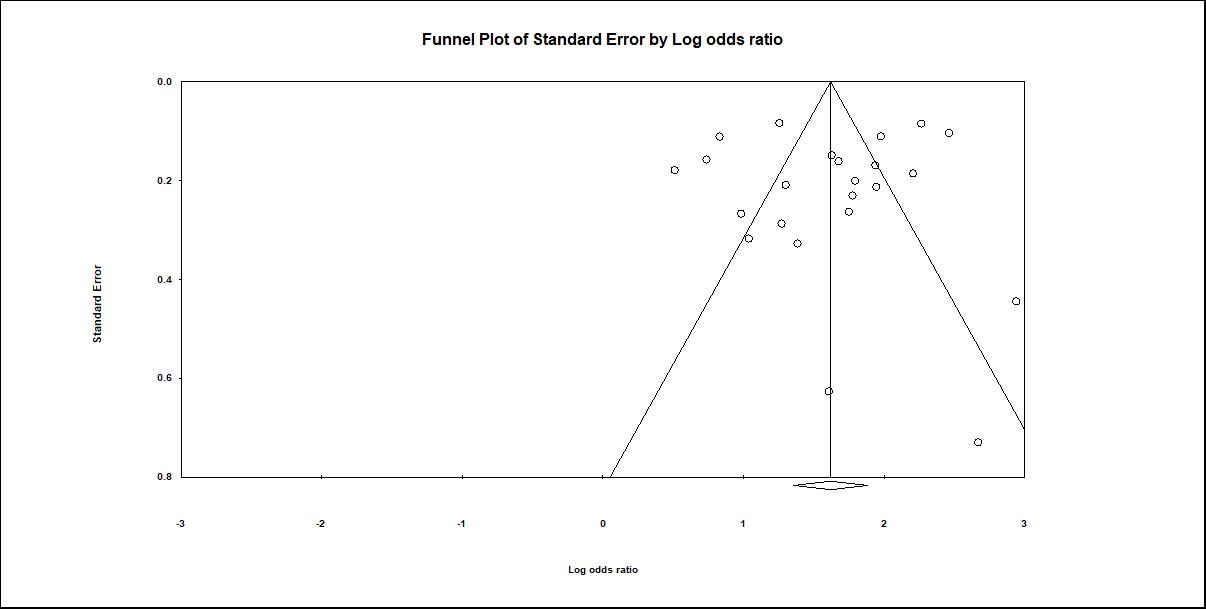

Supplement: Supplementary file 1 [file ijerph-21-01583-s001.zip › Figure S6.tiff]

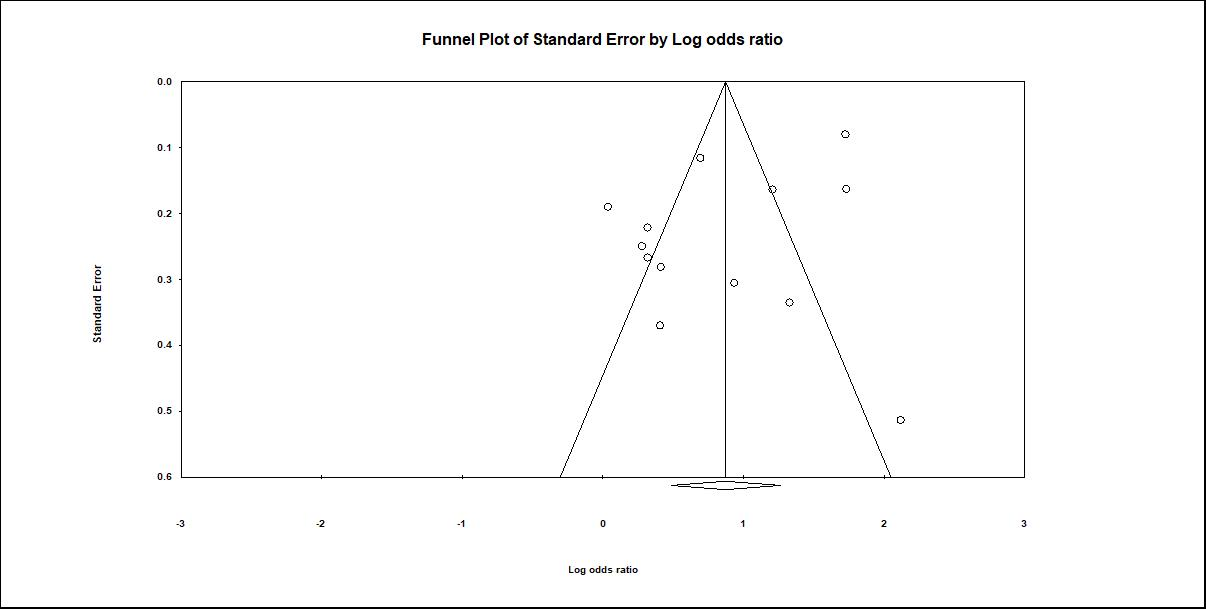

Supplement: Supplementary file 1 [file ijerph-21-01583-s001.zip › Figure S7.tiff]

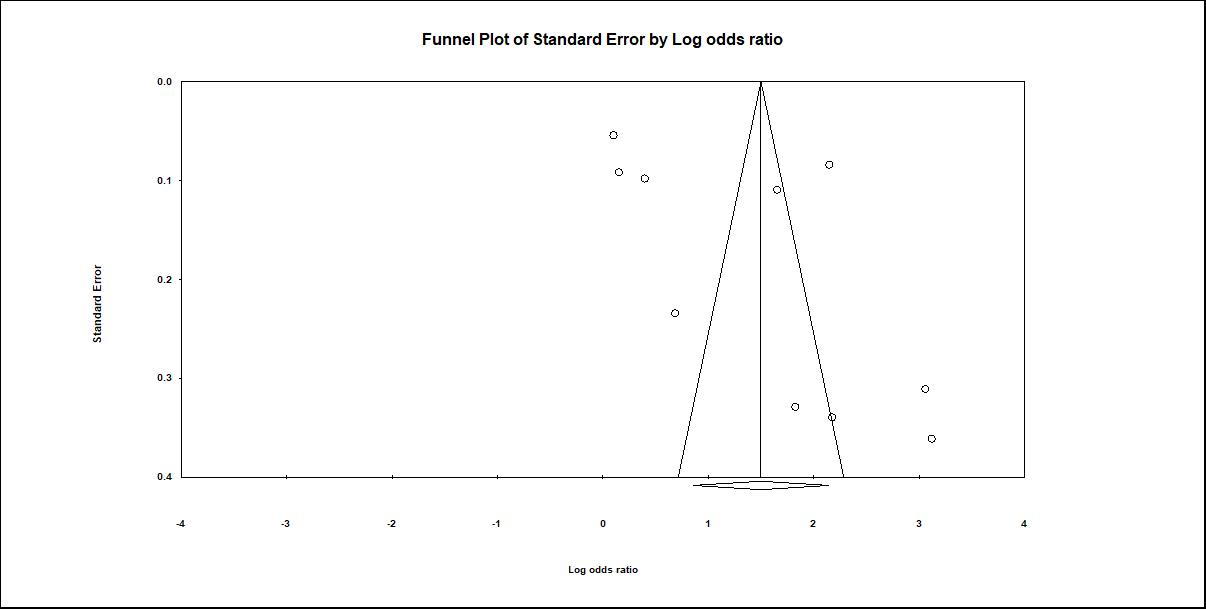

Supplement: Supplementary file 1 [file ijerph-21-01583-s001.zip › Figure S8.tiff]
